# ETV1 Couples Reciprocal FGF23 and PTH Signaling to Renal *CYP24A1* Regulation

**DOI:** 10.21203/rs.3.rs-10474610/v1

**Published:** 2026-08-06

**Authors:** Emmanuel Solis, Kayleigh N. Jennings, Lainey M. Hibbard, Malgorzata M. Kamocka, Sheng Liu, Jun Wan, Seong Min Lee, Jordan M. Towne, Mark B. Meyer, Kenneth E. White

**Affiliations:** 1 Department of Medical and Molecular Genetics, Indiana University School of Medicine, Indianapolis, IN; 2 Department of Medicine/Division of Nephrology and Hypertension, Indiana University School of Medicine, Indianapolis, IN; 3 Department of Nutritional Sciences, University of Wisconsin-Madison, Madison, WI;

**Keywords:** *Cyp27b1*, *Cyp24a1*, VDR, FGF23, ETV1, 1,25(OH)_2_D_3_, calcitriol, COP1

## Abstract

Active vitamin D [1,25(OH)_2_D_3_; 1,25D] synthesis is finely balanced in kidney proximal tubules by the catabolic enzyme CYP24A1 and anabolic CYP27B1. Fibroblast growth factor 23 (FGF23) and 1,25D lower 1,25D by increasing *CYP24A1* and suppressing *CYP27B1*, opposing the actions of parathyroid hormone (PTH). Following FGF23 injections, scRNAseq identified E twenty-six (ETS) member ETV1 as specifically induced in PT S1-S2 cells with *Cyp24a1*. ETV1 was rapidly recruited to *Cyp24a1* enhancers mediating FGF23 and PTH responses, overlapping with VDR, whereas PTH nearly eliminated ETV1 occupancy (4,790 sites to 22). *In vitro*, ETV1 induced *CYP24A1*, and ETV1-VDR interactions were greatly enhanced by 1,25D. Deletion of *Etv1* from mouse kidney epithelium caused FGF23 resistance with elevated iFGF23, and a PTH mimetic salt-inducible kinase inhibitor enhanced COP1-mediated ETV1 degradation. Together, ETV1 is identified as a mediator of reciprocal FGF23 and PTH control of renal vitamin D metabolism, revealing novel mechanisms governing endocrine mineral homeostasis.

## Introduction

Proper control of circulating concentrations of the active form of vitamin D, 1,25(OH)_2_ vitamin D (‘1,25D’), is critical for maintaining normal immune responses, cancer resistance, vascular function, and musculoskeletal health. The systemic balance of 1,25D, as well as phosphate (P) and calcium (Ca^2+^) which are directly and indirectly controlled by this hormone, is coordinated by an exquisitely sensitive, multitissue feedback system. Liver-produced 25-OHD vitamin D serves as the primary substrate for the endocrine-regulated synthesis of 1,25D in the kidney proximal tubule (PT). Fibroblast growth factor-23 (FGF23) is an osteocyte-produced hormone synthesized in response to excess P and 1,25D. In the kidney, FGF23 binds in high-affinity complexes with fibroblast growth factor receptors (FGFRs) and its co-receptor αKlotho (KL), a single-pass transmembrane protein. KL is more highly expressed in the connecting tubule (CNT) and distal convoluted tubule (DCT)^1^, where it has been associated with control of genes regulating serum Na^+^ and Ca^2+^.^2^ However, the overwhelming patient phenotypes in diseases associated with gain or loss of function in FGF23 or KL are due to dysregulated 1,25D and P, which occurs in the kidney PT, thus full understanding of FGF23 function is critical for translational impact for diseases involving FGF23.^3,4^

The key enzymes involved in homeostatic control of vitamin D metabolism are the cytochrome P450 enzymes 24-OHase (CYP24A1) and 1α-OHase (CYP27B1), which are mainly expressed in the kidney and co-localize with KL in the renal PT.^5^ Both *Cyp24a1* and *Cyp27b1* are uniquely and reciprocally regulated by parathyroid hormone (PTH) in response to Ca^2+^ levels, FGF23 in response to P and 1,25D levels, and by 1,25D itself via VDR/RXR binding.^5^ 1,25D also circulates to the intestine to mediate active Ca^2+^ and P absorption. Conditional deletion of KL in the PT has been performed^6,7^, showing elevated 1,25D due to reduced *Cyp24a1* expression and parallel induction of *Cyp27b1*, consistent with loss of FGF23 bioactivity. Outside of the kidney, *Cyp27b1* is regulated by inflammation and *Cyp24a1* is regulated by 1,25D, making the effects of FGF23 and PTH specific to kidney.^5,8^ Thus, FGF23, a peptide hormone, and 1,25D, a steroid hormone, operationally control *Cyp24a1* and *Cyp27b1* expression in the same direction, activating and inhibiting, respectively. However, whether the actions of FGF23/1,25D and PTH are reciprocal but coupled at the molecular level under normal circumstances, and how diseases affecting these hormones alter PT 1,25D metabolism are completely unknown.

Our previous work has defined important tissue-specific enhancers controlling both *Cyp24a1* and *Cyp27b1*. These enhancers bind a combination of VDR and/or phosphorylated cyclic AMP response element-binding protein (pCREB) in response to either 1,25D or PTH signaling.^9–11^ We defined three regions of *Cyp24a1* genomic control: *the proximal promoter* (‘PRO’), *downstream region 1* (‘DS1’), and *downstream region 2* (‘DS2’) that are conserved in humans.^10^ Both the PRO and DS2 regions are active rather ubiquitously in many tissues and cell types like kidney, intestine, spleen, bone, and others.^10^ These regions are responsible for the 1,25D induction of *Cyp24a1* through actions of the VDR, which were mapped in several prior studies.^10,12^ The DS1 region, however, was only found to have accessible chromatin in PT-S1 and PT-S2 cells^5^, conferred activity of PTH suppression, and was also responsible for FGF23 induction of *Cyp24a1*. We defined these activities by extensive histone marker studies and CREB binding for PTH, however as the PT-specific TF for FGF23 activity was unknown.^10^ When the DS1 region was CRISPR-deleted from mice (C24-ΔDS1 mice), there was a reduction in basal expression of *Cyp24a1*, a reduction in FGF23 induction, and elimination of PTH suppression^10^, underscoring the critical nature of genomic control of 1,25D synthesis. Further, it was determined that FGF23 may be a major driver of the basal *Cyp24a1* levels, as deletion of DS1 reduced basal expression, demonstrating the critical role for the kidney eliminating excess 1,25D to maintain this balance. Powerful genetic studies determined that although *Cyp27b1* is required for producing 1,25D, the necessary balance of maintaining vitamin D metabolism relies more on catabolic *Cyp24a1* expression as the critical factor controlling circulating 1,25D.^8,13^ On both *Cyp24a1* and *Cyp27b1*, increased genomic occupancy of VDR or pCREB coincides with increased transcriptional activation^9,10,14^, however the mechanisms of suppression were not explained as easily through removal of these TFs and thus, these critical control mechanisms have remained elusive.

Chronic kidney disease (CKD; affecting approximately 1 in 7 in the US), significantly disordered mineral metabolism and reductions in systemic 1,25D occur after the dramatic downregulation of renal KL, as shown in mouse models and patients.^15–17^ FGF23 binding to FGFR1/KL stimulates ERK/MAPK-mediated transcription factor (TF) early growth response gene-1 (EGR1), which is virtually diagnostic for FGF23/KL bioactivity, as well as almost all paracrine FGF-FGFR interactions.^18^ Using global *Egr1*-KO mice, this TF has been genetically linked to control of P resorption via NPT2A in the renal PT.^19^ However, the ablation of *Egr1* in mice did not affect transcriptional changes in *Cyp24a1*^19^, supporting that other unique cellular components link FGF23 to the control of 1,25D. ERK/MAPK is required for *Cyp24a1* mRNA expression, as demonstrated by PMA-activated ERK/MAPK and 1,25D induced synergism, however the mechanisms responsible for this effect are unknown.^20^ An ETS binding site (EBS) was identified near the CYP24A1 promoter adjacent to the known VDREs based on sequence analysis.^21,22^ However, with the known overlapping binding specificities throughout the ETS family, the exact identity of the critical member controlling CYP24A1 *in vivo* could not be identified. ETS transcription factors are characterized as signal-regulated transcription factors (SRTFs), which control the ultimate transcriptional output of cascades related to the regulation of critical metabolic processes and are also known to serve important roles in kidney development, mitochondrial biogenesis, and acute kidney injury.^23–25^ The ETS PEA3 sub-family members ETV1, −4, −5 have a more conserved ETS binding motif with transactivation domains within the *N-* and *C*-termini.^26,27^ This sub-family has shown significant importance in endocrine signaling, as stability of PEA3 members led to decreased β-cell insulin secretion.^28^ The role of the ETV family TFs in kidney hormone-mediated mineral handling has remained unexplored until our studies.

Herein, we identified ETV1 as a PT-localized TF stimulated by FGF23, that undergoes enhanced function in the presence of 1,25D, and is reciprocally inhibited by PTH. ETV1 genomic binding is activated by FGF23 and inhibited by PTH, which directly and almost completely removes ETV1 from the genome. Surprisingly, ETV1 directly interacts with VDR, and both 1,25D and FGF23 have synergistic activity in regulation of *Cyp24a1*. This system is regulated via proteasomal degradation, a novel concept in phosphate and vitamin D metabolism. Due to the powerful systemic actions of 1,25D, and high sensitivity of FGF23 production in response to 1,25D, these collective data support a new homeostatic model where the dual actions of 1,25D via VDR, and FGF23 via ETV1, work directly and conjointly to regulate *Cyp24a1*, exquisitely controlling kidney 1,25D synthesis.

## Results

### FGF23-dependent PT-specific transcriptional reprogramming revealed unique changes in ETV1

**Fig. 1:**
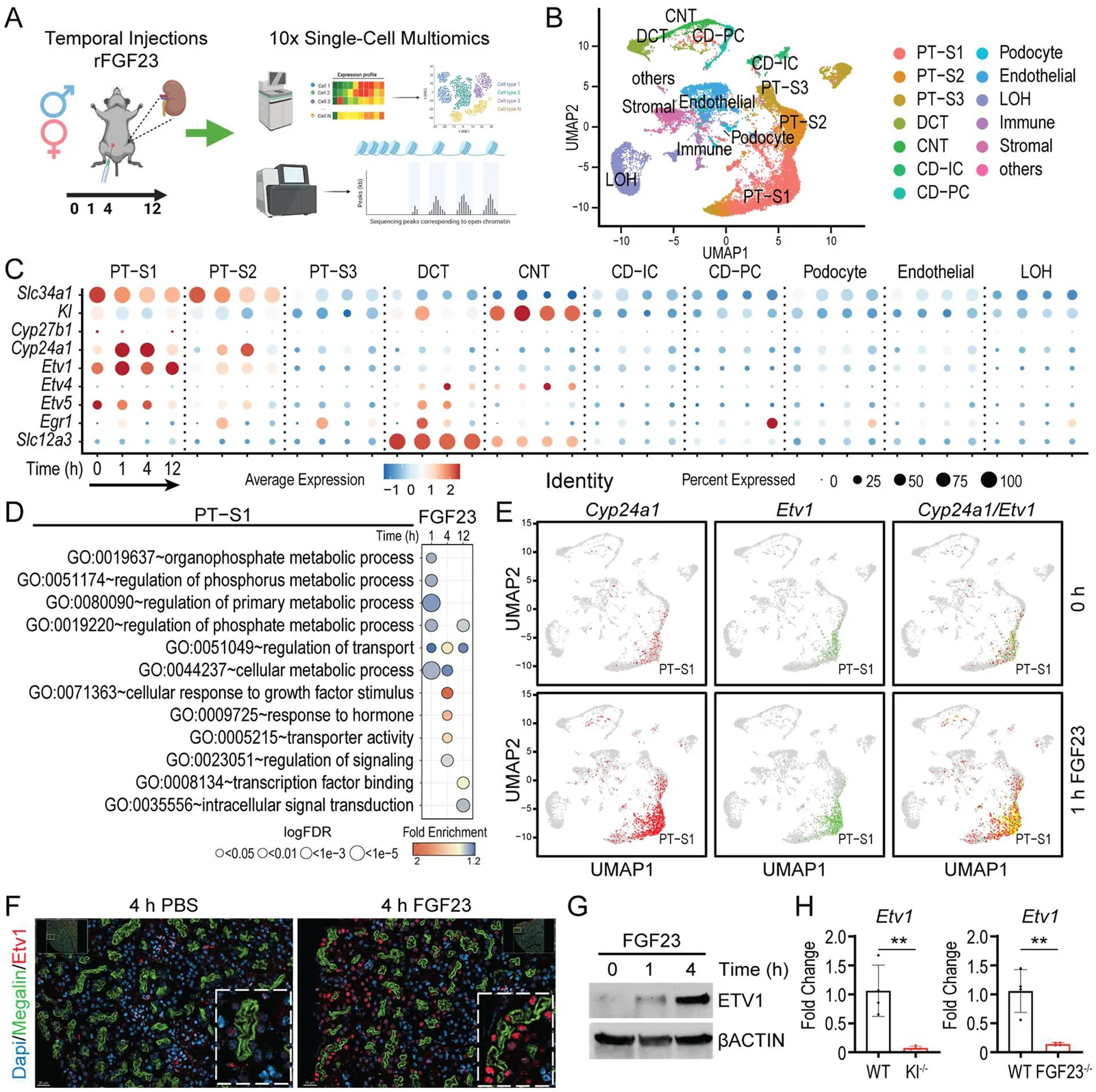
FGF23 bioactivity drives PT ETV1 expression. **A**, experimental overview. Pooled male and female kidneys from FGF23-injected mice (250 ng/g bw for 0, 1, 4, or 12 h) were dissociated into single-cell (sc) suspensions followed by tissue processing and 10X Multiome (scRNAseq and scATACseq from the same cell), followed by downstream computational and molecular analysis. **B**, 10X Multiome UMAP plots of analysis of scRNAseq and ATACseq identified the renal cell population clusters. **C**, dot plot of nephron epithelial cell populations and relative expression of key genes, FDR<0.05 for *Cyp24a1, Cyp27b1, Etv1, Egr1*. **D**, Gene Ontology (GO) pathway enrichment analysis of representative pathways significantly enriched by FGF23 administration with time (1, 4, 12 h versus 0 h; p<0.05 – p<1e-5). **E**, feature plot of co-localized *Cyp24a1* (red) and *Etv1* (green) mRNAs and co-localized expression in PTS1 cells (yellow) at 0 and 1 h. **F**, immunofluorescence analysis of nuclear ETV1 (red) colocalized with DAPI (blue) in megalin^+^ (green) PT segments after 4 h injections of vehicle (PBS; *left image*) or FGF23 (*right image*) into normal mice. Insets: low power kidney image with region of interest boxed; dashed box: enlarged individual PT segments; scale bar lower left. **G**, immunoblot of kidney nuclear ETV1 protein as compared to β-ACTIN at time 0 h, and 1 and 4 h post-FGF23 injection. **H**, kidney *Etv1* mRNA vs *β-actin* in *Kl*-KO and *Fgf23*-KO mice; n=4, and comparing WT to *Fgf*23-KO or *Kl*-KO unpaired T-test, **, p <0.01).

To undertake unbiased identification of the mechanisms controlling FGF23 bioactivity in the kidney that specifically localized with kidney proximal tubule (PT) genes known to carry out critical mineral metabolic processes, 10X Multiome (scRNAseq and scATACseq from the same cell) was performed on kidneys from mice temporally injected with FGF23. In this regard, wild type (WT) male and female mouse pairs (10 wks of age) were injected at 0, 1, 4, and 12 h with FGF23 (250 ng/g bw), and kidneys were isolated, and pooled male and female nuclei prepared for sequencing library assembly (Fig. 1A). Sex-specific nephron segment cell populations and immune cells were segregated using *Xist* (X chromosome) and Y chromosome markers (Supplemental Fig. 1, according to ref.^29^); male represented 11,153 total cells, and females, 15,982 cells. Unsupervised clustering analysis identified cell populations representative of major renal cell types defined through their unique transcriptomic signatures, with sex-specific PT-S3 regions^30,31^ (UMAP analyses; Fig. 1B). Upon analysis of differentially expressed genes (DEG), there were no sex differences with FGF23 treatment, thus the mice were analyzed together, and as expected, FGF23 injections also did not induce significant changes in cell population clustering (Supplemental Fig. 2).

To identify transcriptional and genomic changes with FGF23 delivery over time, DEG analysis and differential accessible regions (DAR) were performed across timepoints (1, 4, 12 h). Sodium-phosphate cotransporter *Slc34a1* (NPT2A; PT-specific marker) and the sodium channel *Slc12a3* (distal tubule: ‘DT’; DCT and CNT) were appropriately detected and thus served as positive controls for these distinct cell populations (Fig. 1C). *KL* was confirmed as modestly expressed in PT and higher in DT cells. We also found that the FGF23-responsive gene *Egr1* was appropriately and acutely increased at 1–4 h, and tracked with *KL* in both PT and DT segments (Fig. 1C). In contrast, PT-S1/S2 specific changes were also identified, with the ETS TF *Etv1* being present in a higher proportion of PT-S1/S2 cells at baseline (0 h), then significantly upregulated in response to FGF23, coinciding with elevated expression of *Cyp24a1* and reduced *Cyp27b1* in the PT-S1/S2 (Fig. 1C; p<0.05). Although *Etv1* was modestly detectable in the renal DCT and CNT segments, *Etv1* did not undergo significant induction at these sites following FGF23 administration (Fig. 1C). ETV1, −4, and −5 are frequently studied together as they have been found to have overlapping activities^32–36^. Here, in contrast to *Etv1*, *Etv5* mRNA was present in both PT and DT segments, but was slightly reduced with FGF23 administration, and *Etv4* was localized primarily in distal nephron cell populations (Fig. 1C). Further, pathway analysis revealed significant upregulation of gene sets related to phosphate metabolism, hormone signaling, and renal transport (Fig. 1D), all consistent with known actions of FGF23 *in vivo*.^37^ The strong co-localization of *Etv1* and *Cyp24a1* mRNAs was also confirmed (Fig. 1E), whereas *Etv5* showed far lower conjoint expression with *Cyp24a1* (Supplemental Fig. 3). Considering: 1) the precise basal expression of PTS1-S2 *Etv1 mRNA*, 2) strong PT-localized induction of *Etv1* by FGF23, and 3) coordinate overlap of *Etv1* with *Cyp24a1* expression, we focused our studies on *Etv1*.

To confirm the regulation of *Etv1* by FGF23 *in vivo*, we performed histological/immunofluorescent (IF) analyses after saline (Fig. 1F, *left*) or FGF23 (250 ng/kg bw) injections (4h, *right*) into normal mice as above. Compared to control, FGF23 delivery increased reactive ETV1 protein (*red staining*, Fig. 1F) that co-localized with DAPI (*blue stain*) in the nucleus of Megalin^+^ PT epithelial cells of the outer kidney cortex (*green staining*, Fig. 1F; *inset:* low power). Non-epithelial cells such as glomerular cells, lacked FGF23-inducible ETV1 staining. Kidney nuclear fractions were also isolated following similar FGF23 injections as above, and upon immunoblot, compared to mice at baseline (saline, 4 h), ETV1 showed nuclear localization at 1 h, which was enhanced at 4 h (Fig. 1G). ETV1 transcripts were next assessed in total kidney from both *Fgf23*-KO and *KL*-KO mice to test the ‘converse’ situation of ablated FGF23 bioactivity, which showed almost completely suppressed (>90%) *Etv1* mRNAs compared to littermate WT controls (p<0.001) (Fig. 1H). These data support that *Etv1* is a PT-localized TF and potently induced at the transcriptional and translational levels by FGF23 activity, which may also be required to maintain basal PT *Etv1* expression.

### ETV1 ChIP-seq shows binding in critical *Cyp24a1* regulatory loci *in vivo*

**Fig. 2.**
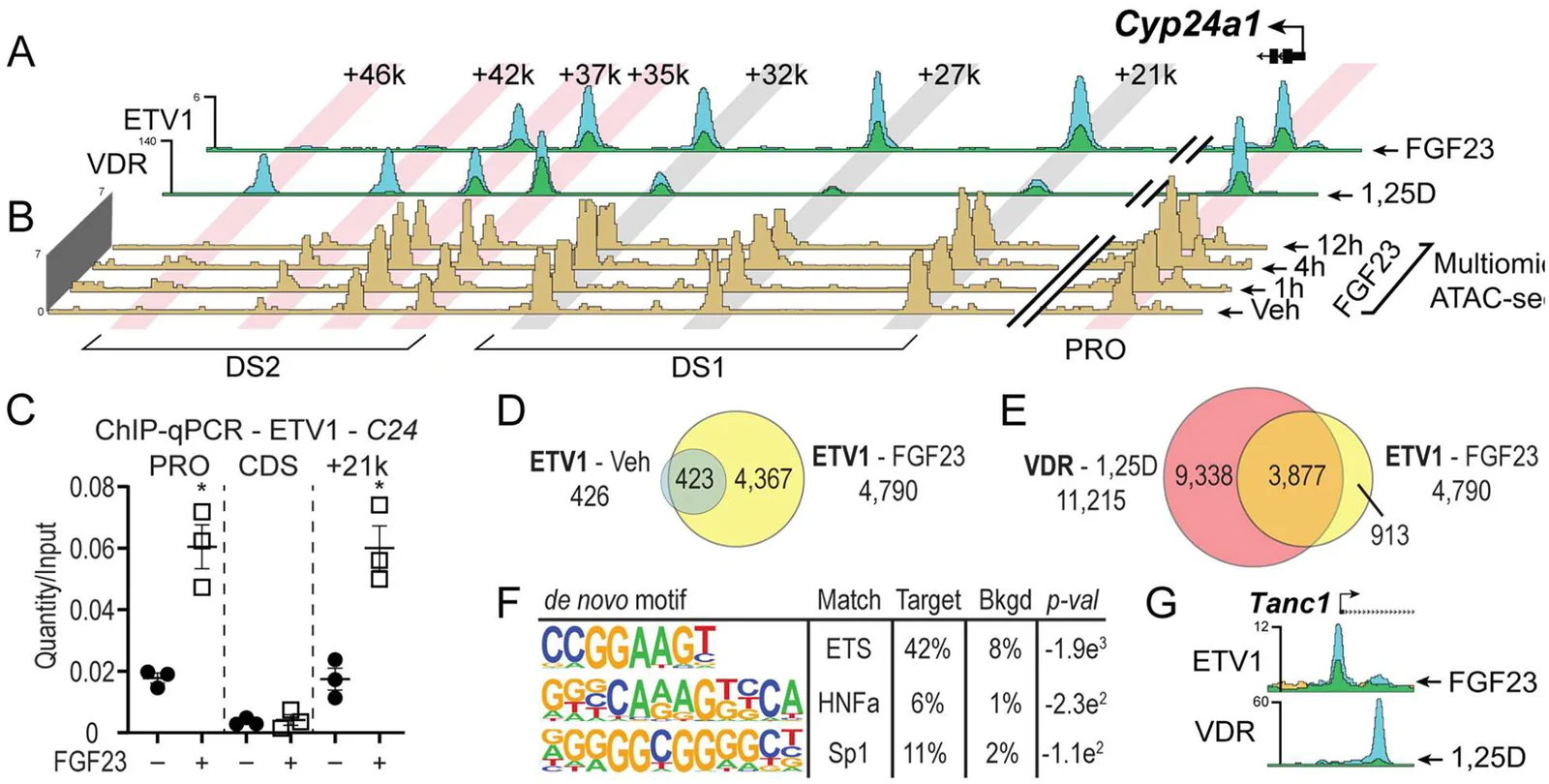
ChIP assays link ETV1 with VDR to critical PT 1,25D metabolizing *Cyp24a1 in vivo*. **A**, ChIP-seq at the *Cyp24a1* locus for ETV1 and VDR following 1,25D (10 ng/g bw) or FGF23 (50 ng/g bw) injections for 1 h versus vehicle (PBS/EtOH) into 8–10 w old male C57BL/6J mice (n=3). Normalized and triplicate averaged ChIP-seq data tracks displayed in the UCSC genome browser (mm9) alongside our prior studies with 1,25D treated kidney samples^10,14^ as overlaid, stacked data tracks (veh: *yellow*; treatment: *blue*, overlapped track signal: *green*). Genomic regions displayed are indicated for each region and maximum height of tag sequence density for each data track indicated on the Y-axis (normalized to input and 10^7^ tags). Enhancers are highlighted and color coded with the red regions as tissue ubiquitous enhancers and gray regions as PT specific enhancers^5,10^. The *Cyp24a1* locus was truncated between PRO and +21k for brevity^10,14^. **B**, Multiome scATAC-seq at 0, 1, 4, 12 h post FGF23 treatment for *Cyp24a1*. **C**, qPCR analysis of ChIP DNA with primers at *Cyp24a1* PRO, *Cyp24a1* CDS, and *Cyp24a1* +21k regions. *, p < 0.05 paired t-test. **D**, Venn diagrams of ETV1 ChIP-seq peaks in vehicle (Veh) and FGF23 treated conditions. **E**, Venn diagrams of FGF23-ETV1 and 1,25D-VDR ChIP-seq peaks. **F**, binding site logo motifs for overrepresentation of short motifs (8–10 nt) in the ETV1 peaks (4,790). **G**, ChIP-seq data surrounding the *Tanc1* gene promoter with same details as in (A).

FGF23 specifically induced ETV1 in the renal PT, the primary site of active 1,25D synthesis, therefore we next tested the ability of ETV1 to bind to genomic regions of the *Cyp24a1* loci, by performing ChIP-seq from mouse kidney *in vivo*. In this regard, C57BL/6J male mice (n=3 per treatment) were administered vehicle, 50 ng/g bw recombinant mouse-FGF23 for ETV1 (1 h), or 10 ng/g bw 1,25(OH)_2_D_3_ for VDR (1 h, VDR data from^38^) as indicated prior to ChIP-assay (Fig. 2A). The 1 h time point was previously optimized for FGF23 activity by histone markers^10^ and coincides with detectable increases in nuclear ETV1 protein (Fig. 1G). As seen in Fig. 2A, ETV1 bound in the *Cyp24a1* PRO region, DS1 region, and part of the DS2 region. Not only was ETV1 bound to these regions, but occupancy was markedly and significantly increased after FGF23 treatment in both gene loci (increased blue signal, p < 0.05 in PRO, +21k, +27k, +32k, +35k, +37k, statistics in Supplemental Table 1). ETV1 was found bound to most, but not all (+42k, +46k) 1,25D-stimulated VDR-bound regions (Fig. 2A) in the *Cyp24a1* locus. The scATAC-seq from our Multiome data following temporal FGF23 injections at 0, 1, 4, and 12 h (from Figs. 1A–C) aligned with the enhancers for *Cyp24a1* which indicate that these regions are already accessible at baseline (vehicle) and likely occupied by ETV1 or other transcription factors (Fig 2B). The chromatin accessibility was maintained throughout FGF23 treatments.

We confirmed the binding increase at the *Cyp24a1* PRO and +21k regions by ChIP-qPCR (Fig. 2C). Genome-wide analyses found 4,790 binding peaks (Supplemental Table 2) for ETV1 in mouse kidney after FGF23 treatment that was increased from 426 basal peaks (Vehicle, Veh) (Fig. 2D). ETV1 and VDR occupied many of the same genomic regions (Fig. 2E). In this regard, a genome-wide overlap of the VDR sites (after 1,25D treatment^11^) and the ETV1 sites (after FGF23 treatment) show that 3,877 of the 4,790 ETV1 peaks overlap with 1,25D-activated VDR. A *de novo* motif analysis (Fig. 2F) demonstrated that an ETS-like binding site was found to be the top enriched motif compared to background (42% vs 8% bkgd, p=−1.9e^3^) indicating our ChIP was specific. Despite the large overlap of VDR and ETV1 sites, there were many regions where ETV1 binds without VDR such as near the promoter of *Tanc1* (a scaffold protein^39^; upregulated by FGF23) as seen in Fig. 2G.

### ETV1 interacts with VDR in the regulation of *CYP24A1 in vitro*

**Fig. 3.**
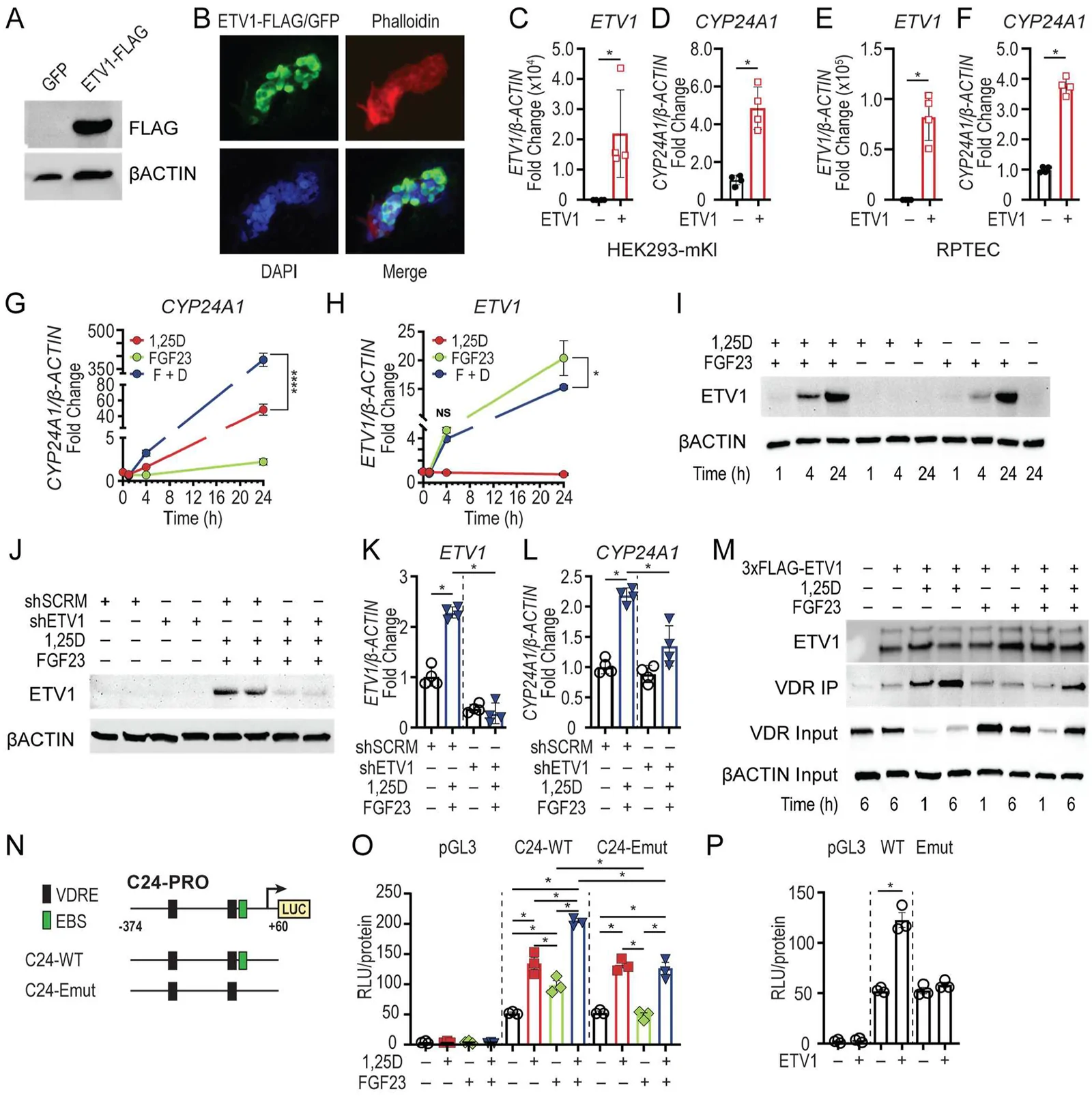
FGF23- and 1,25D/VDR-mediated ETV1 functional interactions. **A**, immunoblot of HEK-mKL cell lysates transfected with a human ETV1-FLAG-GFP cDNA for 24 h and tested for expression versus its GFP-backbone negative control as compared to β-ACTIN, using an anti-FLAG(M2) antibody; **B**, immunofluorescence of HEK-mKL cells transfected with the ETV1 cDNA using direct GFP fluorescent co-localization (green), phalloidin to mark the cell body (red), as well as DAPI (blue), and images merged (Merge). Levels of *ETV1* mRNA (**C**) and *CYP24A1* mRNA (**D**) in HEK-mKL cells; and *ETV1* mRNA (**E**) and *CYP24A1* mRNA (**F**) in RPTEC cells transfected with ETV1-GFP cDNA (*, p<0.05 by unpaired T-test). Expression of *CYP24A1* mRNA (**G**) and endogenous *ETV1* mRNA (**H**) in HEK-mKL cells treated with Veh (PBS/EtOH), human FGF23 (50 ng/mL), 1,25D (10 nM), or FGF23+1,25D for 0, 1, 4, and 24 h. Data are displayed as fold change versus *β-ACTIN* mRNA. Data were compared by 1-way ANOVA with tukey post-tests comparing treatments to each other, *, p < 0.05 or ****, p <0.001 as indicated. **I**, immunoblot of native ETV1 protein induction in HEK-mKL cells after treatment with FGF23 (50 ng/mL), 1,25D (10 nM), and FGF23+1,25D for 1, 4, and 24 h compared against β-ACTIN. **J**, immunoblot of HEK-mKL cells transduced with a lentiviral scrambled control short hairpin (sh)RNA (shSCRM) or shRNA to ETV1 (shETV1) for 5 d, followed by 4 h of treatment with 50 ng/mL FGF23 and/or 10 nM 1,25D; protein was tested in duplicate compared against β-ACTIN. Gene expression was tested for *ETV1* (**K**) and *CYP24A1* (**L**) by qPCR using the same experimental conditions in (J) n=4 (*p<0.05 versus shSCRM controls). **M**, immunoblot of immunoprecipitations (IP) of ETV1-FLAG plasmids transfected into HEK-mKL cells for 24 h then treated with vehicle, or FGF23 and/or 1,25D as above for 1 or 6 h; immunoblot for ETV1 and VDR (‘VDR-IP’) was performed; VDR protein in the depleted cell lysates post-IP are also shown (‘VDR Input’) compared to β-ACTIN (‘βACTIN Input’). **N**, schematic of luciferase reporters at the *Cyp24a1* PRO region. **O**, HEK-mKL cells were transfected with pGL3basic-luc(empty), C24-WT, or C24-Emut and treated for 24 h with vehicle (V, PBS/EtOH), FGF23 (50 ng/mL), 1,25D (10 nM), or FGF23+1,25D in triplicate (n=3) and luciferase values were measured. Luciferase values were normalized by total protein. Data were compared by 2-way ANOVA between treatment and construct with Tukey post-tests comparing treatments to each other, *, p < 0.05 as indicated. **P**, the ETV1-FLAG cDNA was cotransfected with pGL3-luc (empty), C24-WT, or C24-Emut constructs. Samples and stats were processed as in **O**.

To test whether ETV1 controlled *CYP24A1*, a human *ETV1*-FLAG tagged cDNA (with GFP reporter) or GFP cDNA alone, were transiently transfected into human embryonic kidney (HEK293) cells expressing membrane KL (HEK-mKL) cells.^40^ ETV1-FLAG protein expression confirmed by immunoblot with anti-FLAG (Fig. 3A), as well as IF (GFP), cytoskeleton - phalloidin (red) (Fig. 3B). Compared to control GFP-transfected cells, *ETV1* cDNA-transfected cells had confirmed increases in *ETV1* mRNA (Fig. 3C) and also upregulated *CYP24A1* mRNA (Fig. 3D), which was replicated in the human RPTEC proximal tubule cell line (Fig. 3E and 3F). Overexpression of an ETV5 cDNA construct did not increase *CYP24A1* expression *in vitro* (Supplemental Fig. 4). We recently demonstrated that 1,25D and FGF23 act synergistically *in vivo*, with cotreatment of both hormones increasing the transcriptional activity of *Cyp24a1* and suppressing *Cyp27b1* more than either hormone alone.^38^ HEK-mKL cells were treated with 10 nM 1,25D, 50 ng/mL human FGF23, or both hormones for 0, 1, 4, and 24 h. As seen in Fig. 3G, we found that *CYP24A1* mRNA was increased by 2-fold with FGF23 alone, a 50-fold increase with 1,25D, and co-treatment produced an almost 400-fold increase in expression. *ETV1* mRNA was increased by FGF23 by 20-fold by 24 h, however, *ETV1* was not increased by 1,25D, either alone, or in combination with FGF23 (Fig. 3H). Further, as assessed by immunoblot, ETV1 protein was increased with FGF23 alone and with FGF23 + 1,25D (4 and 24 h; Fig. 3I), and similar to transcription (see Fig. 3C), 1,25D (10 nM) alone had no effect on ETV1 protein (Fig. 3I). HEK-mKL cells were next transduced with shRNA lentivirus for either a scramble sequence (Scrm) or ETV1 sequence (n=4); cells were treated with FGF23 + 1,25D as above for 4 h and protein tested by immunoblot (Fig. 3J) and mRNA expression of *ETV1* (Fig. 3K) and *CYP24A1* (Fig. 3L). In confirmation of our model, shRNA knockdown of ETV1 reduced FGF23-mediated *CYP24A1* expression (Fig. 3L).

Our *in vivo* ChIP data showed a strong genomic correlation between VDR and ETV1 occupancy sites, and both *in vivo* and *in vitro* cotreatment of 1,25D and FGF23 cause a synergistic increase of *CYP24A1*. Therefore, we next tested for direct ETV1-VDR interactions in Fig. 3M. Importantly, only in cells treated with 1,25D (10 nM, 1 or 6 h) or FGF23+1,25D could VDR and ETV1 be co-precipitated to a level above cells treated with vehicle (Veh) or FGF23 alone, supporting that although 1,25D does not independently drive ETV1 protein increases (see Fig. 3I) it markedly enhanced ETV1-VDR interactions (Fig. 3M). Thus, these data support that FGF23 and 1,25D act synergistically to control *CYP24A1*, and ETV1 directly interacts with VDR, consistent with co-regional isolation of ETV1 and VDR in *Cyp24a1* by ChIP-seq.

Finally, we found that ETV1 binds to all the crucial *Cyp24a1* enhancers (Fig. 2A) in both the PRO and DS1 regions to confer FGF23 activity both *in vivo* and *in vitro*. We next wanted to test sequence specificity of ETV1 binding to activation of the *Cyp24a1* gene and the synergistic relationship with VDR. We created luciferase reporters to test these interactions in the HEK-mKL cells in the mouse *Cyp24a1* PRO region. These mutation constructs are shown schematically in Fig. 3N with VDREs (black) or EBS (green) indicated. These sequences were cloned into the pGL3-luc plasmid, transfected into the human HEK-mKL cell line, and treated with vehicle, 1,25D (10 nM), FGF23 (50 ng/mL), or both 1,25D + FGF23 for 24 h as indicated on the X-axis of Fig. 3O. It was found that the C24-WT construct behaved identically to the native *Cyp24a1* gene expression with an increase by FGF23 treatment and 1,25D alone, as well as a synergistic increase of the 1,25D + FGF23 treatment group. Additionally, the human ETV1-GFP cDNA was also transfected in Fig. 3P. The C24-WT baseline activity was increased; however with mutation of the EBS in the C24-Emut, this increase is ablated (Fig. 3P). These data point to the EBS as critical for the actions of FGF23 on the *Cyp24a1* PRO reporter construct.

### Targeting kidney *Etv1 in vivo* disrupts FGF23 responsiveness and vitamin D metabolism

*Etv1* is critical for neonatal tissue development,^36,41^ therefore we undertook an inducible-deletion strategy to test the genetic loss of ETV1 function on PT mineral metabolism. To conditionally delete *Etv1* from kidney, the flox-*Etv1* allele was bred onto the doxycycline (DOX)-inducible *Pax8*-rtTA/LC1-Cre (herein referred to as ‘*Pax8*-Cre’; targets nephron epithelial cells)^42^.

**Fig. 4:**
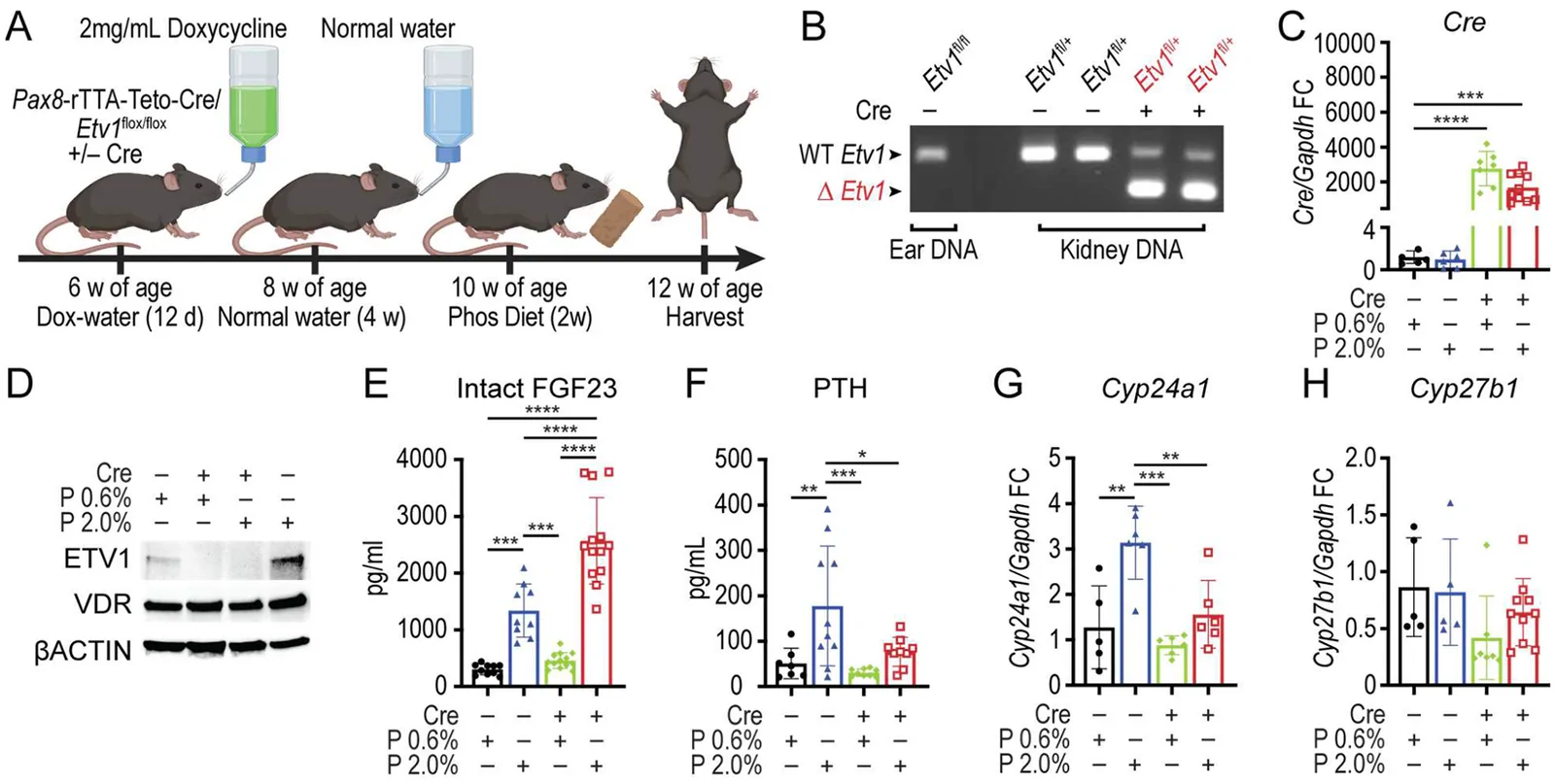
Mice with conditional deletion of *Etv1* from kidney are resistant to FGF23. **A**, experimental overview: *Etv1*^*fl*/fl^/*Pax8*-Cre^+/−^ mice (6–7 w of age) were provided DOX (12 days) in their drinking water followed by a washout period (4 w) and experimental diets (2 w), then euthanasia at 12 w of age. **B**, allele-specific PCR for DOX-inducible *Pax8*-Cre mediated *Etv1*^fl/fl^ allele recombination in DNA from kidney (‘ΔETV1’) compared to ear notch. **C**. *Cre* mRNA was elevated in the *Etv1*^fl/fl^-Cre^+^ mice versus *Etv1*^fl/fl^-Cre^−^ mice (p<0.01). **D**, nuclear ETV1 protein was examined following DOX treatment, in *Etv1*^fl/fl^-Cre^+/−^ mice receiving normal (0.6%) or high (2.0%) phosphate (P) diet by immunoblot. **E**, plasma intact (i)FGF23 measured by ELISA in *Etv1*^fl/fl^-Cre^−/+^ mice (n=9–13/group). **F**. plasma PTH as measured by ELISA in *Etv1*^fl/fl^-Cre^−/+^ mice (n=7–9/group). Kidney expression of *Cyp24a1* **(G)**, and *Cyp27b1* **(H)** mRNAs versus *Gapdh* mRNA from the diet-treated *Etv1*^fl/fl^/*Pax8*-Cre^+/−^ mice as determined by qPCR (n=6–10/group). Data for C, E-F were compared by Welch s t-test or 2-way ANOVA between diet and genotype with Tukey post-tests comparing treatments to each other, *, p <0.05, **, p < 0.01, ***, p < 0.005, ****, p < 0.0001 as indicated.

To determine the effects of loss of *Etv1*, *Etv1*^fl/fl^-Cre^+/−^ mice (6 w of age, n=4–5 male and female/group) were provided DOX (n=4–5 male and female/group; 2 mg/ml in drinking water) for 12 days, followed by a 4 w recovery period (Fig. 4A). Mice were also challenged with a normal (0.6%) or high phosphate (2%) diet for the last 2 w of the recovery period (Fig. 4A). The flox-*Etv1* allele was successfully recombined in the kidneys of the *Etv1*^fl/fl^-Cre^+^ mice as shown by recombination-specific PCR (‘Δ*Etv1*’; Fig. 4B). Cre mRNA was induced in the mice receiving DOX (Fig. 4C) and nuclear ETV1 protein was only induced in the *Etv1*^*fl*/fl^-Cre^−^ mice receiving the high phosphate diet as determined by immunoblot (Fig. 4D), confirming Cre-mediated recombination. The *Etv1*^fl/fl^-Cre^−^ mice increased iFGF23 plasma concentrations during high phosphate as expected, and the *Etv1*^fl/fl^-Cre^+^ showed even greater elevation of iFGF23 during high phosphate administration (Fig. 4E). Further, in response to high phosphate diet the *Etv1*^fl/fl^-Cre^−^ mice increased PTH, but the *Etv1*^fl/fl^-Cre^+^ were resistant to this induction (Fig. 4F). Similarly, the *Etv1*^fl/fl^-Cre^−^ mice showed a 4-fold increase in *Cyp24a1* mRNA during challenge, whereas the *Etv1*^fl/fl^-Cre^+^ mice had a significantly blunted response (Fig. 4G); there were no significant changes in *Cyp27b1* mRNA across groups (Fig. 4H), nor was there meaningful diet-induced changes in serum biochemistries (Supplemental Fig. 5). Finally, the influence of reciprocal FGF23 and PTH concentrations was observed as maintaining constant plasma 25D and 1,25D concentrations during treatments (data not shown), highlighting the homeostatic strength of the vitamin D system to correct metabolite and mineral levels. Collectively, these findings demonstrate that *Etv1* is critical for maintaining critical aspects of mineral metabolism, as mice with loss of ETV1 had improper iFGF23 and PTH regulation with corresponding defects in *Cyp24a1* expression, demonstrating the central role of ETV1 in mediating the PT-specific effects of FGF23.

### Reciprocal control of ETV1 by proteosome-mediated turnover

**Fig. 5:**
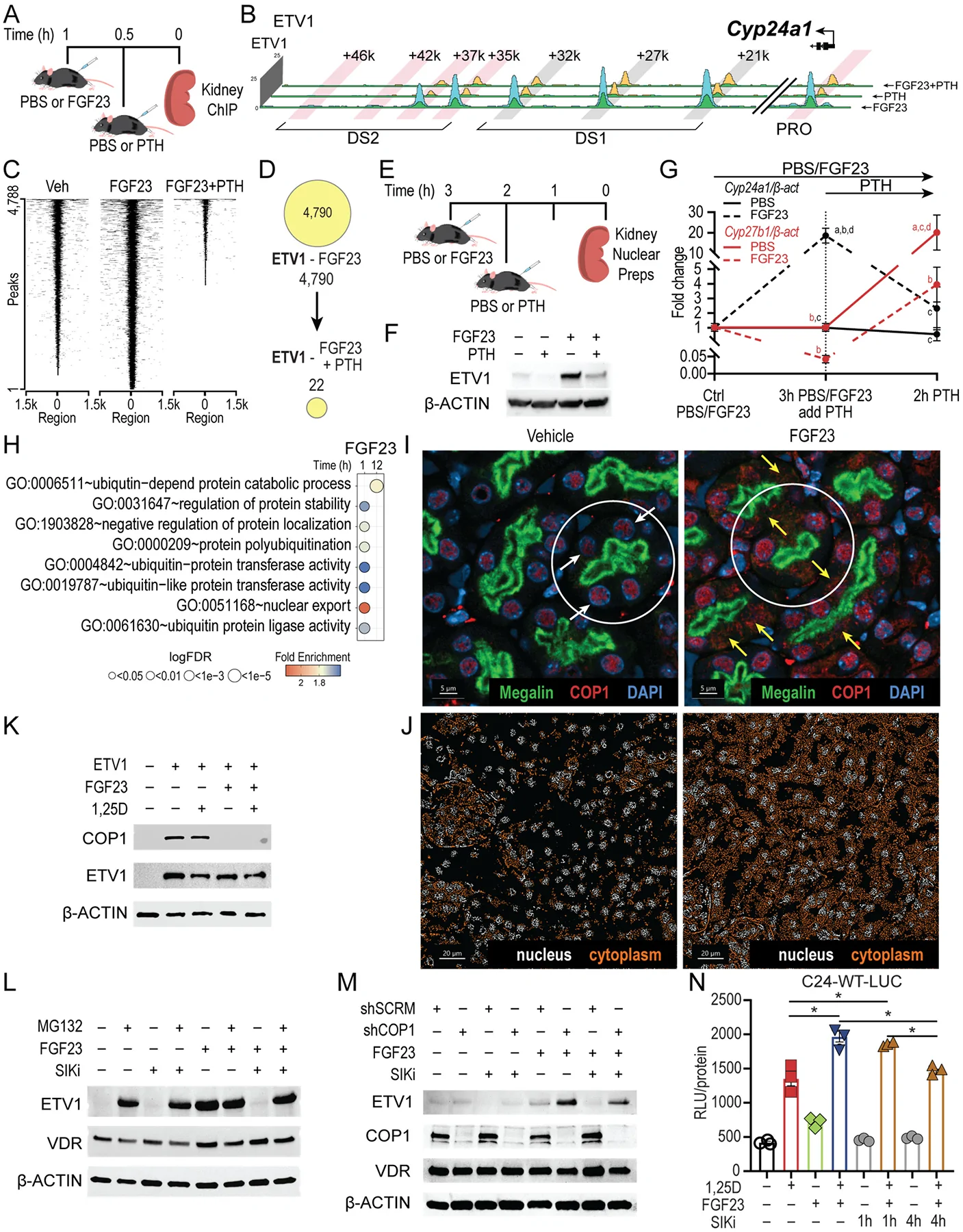
Converse FGF23 and PTH actions on ETV1 via COP1-mediated protein turnover. **A**, Schematic of treatments for mice injected with FGF23 and then PBS or PTH. **B**, ChIP-seq of mouse kidney for ETV1 at the *Cyp24a1* locus after the injections of PTH (230 ng/g bw, 30 min) or FGF23 (50 ng/g bw, 1 h) + PTH for 30 min into 8–10 w old male C57BL/6J mice (n=3) compared to FGF23 alone data (from Fig. 4A). Normalized and triplicate averaged ChIP-seq data tracks displayed in the UCSC genome browser (mm9) as overlaid, stacked data tracks (veh: *yellow*; treatment: *blue*, overlapped track signal: *green*). Genomic regions displayed are indicated for each region and maximum height of tag sequence density for each data track indicated on the Y-axis (normalized to input and 10^7^ tags) as in Fig. 4. **C**, mouse kidney ChIP-seq tag density surrounding the peak regions (±1.5 kb) for ETV1 after vehicle, FGF23, and FGF23+PTH treatments. Increased black intensity indicates increased tag density. **D**, schematic peak regions for ETV1-FGF23 and ETV1-FGF23+PTH treatments. **E**, schematic for PBS and FGF23 treatments and kidney isolation for qPCR and immunoblot. **F**, immunoblot of kidney ETV1 nuclear protein from mice treated with vehicle (PBS), PTH (230 ng/g bw, 2 h), FGF23 (250 ng/g bw, 3 h), or FGF23+PTH compared against β-ACTIN. **G**, qPCR for *Cyp24a1* (black) and *Cyp27b1* (red) during FGF23, PTH, and FGF23+PTH injections (solid lines = relative gene expression for PBS at time 0, then PBS or PTH after 3 h for an additional 2 h as compared to *β-actin* mRNA; dashed lines = relative gene expression for FGF23 at 0 h, then PBS or PTH after 3 h for an additional 2 h as compared to *β-actin* mRNA; ‘a’ indicates significant difference versus ‘PBS 3h/PBS 2h’; ‘b’ indicates significant difference versus ‘PBS 3h/PTH 2h’; ‘c’ indicates significant difference versus ‘FGF23 3h/PBS 2h’; ‘d’ indicates significant difference versus ‘FGF23 3h/PTH 2h’ (p<0.05 as determined by one-way ANOVA). **H**, Multiome Gene Ontology (GO) enrichment analysis of ubiquitination pathways at 1 h in kidney PT-S1 (see Fig. 1). **I**, immunofluorescence of kidney sections from mice injected (4 h) with Vehicle (PBS; *left image*, white arrows delineate nuclear COP1) or 250 ng/g bw FGF23 (*right image*, yellow arrows delineate cytoplasmic COP1); COP1 staining (red), megalin (green), and DAPI (blue); scale shown lower left and circle highlights a tubule comprised of several PT cells. **J**, segmented COP1 signal with color-coded nuclear and cytoplasmic fractions from panel **I** (Vehicle (PBS)-injected mice, *left image*; FGF23-injected mice, *right image*) for nuclear COP1 (white stain) versus cytoplasmic staining (brown). Scale shown lower left. **K**, immunoblot of HEK-mKL cell lysates following ETV1-FLAG cDNA transfection (24 h) then FGF23 (50 ng/mL) with or without 1,25D (10 nM) treatment for 1 h; immunoprecipitations were probed for COP1 and ETV1 compared against β-ACTIN from the original input lysates. **L**, immunoblots of HEK-mKL cell lysates for ETV1 and VDR compared to β-ACTIN following MG132 (5 μM, 2 h), FGF23 (50ng/mL, 4 h), and then SIKi (HG-99101, 5μM for 2 h) as indicated. **M**, immunoblots of HEK-mKL cell lysates for ETV1, COP1, and VDR compared against β-ACTIN after treatment with FGF23 (50 ng/mL; 4 h) with or without SIKi (HG-99101; 5μM for 2 h) as indicated following 5 d pre-treatment with a COP1 short hairpin RNA (shRNA) lentivirus; ‘shCOP1’) or scrambled control shRNA (‘shSCRM’). **N**, HEK-mKL cells were transfected with C24-WT-LUC (from Fig. 3) and treated for 18 h with vehicle (V, PBS/EtOH), FGF23 (50 ng/mL), 1,25D (10 nM), or FGF23+1,25D in triplicate (n=3) with an additional 1 h or 4 h of 5 μM HG-99101 (SIKi). Luciferase values were normalized by total protein. Data were compared by One-way and Two-way ANOVA for treatment and time with Tukey post-tests comparing treatments to each other, *, p < 0.05 as indicated.

The actions of FGF23 to suppress 1,25D are directly opposed by PTH and the mechanisms directing this regulation are unknown. To test whether ETV1 expression is influenced by PTH *in vivo*, WT male mice (n=3) were given an injection of FGF23 (50 ng/g bw) for 30 min, then PTH (230 ng/g) for an additional 30 min (1 h total) or with PTH (230 ng/g bw) for 30 min, and ChIP-seq was performed (Fig. 5A, schematic). We compared the PTH treatment and FGF23+PTH treatment to the FGF23 (Fig. 5B) for ETV1 recruitment to the genome. In the *Cyp24a1* locus, PTH treatment alone, and even PTH after FGF23, not only opposed the ETV1 increases with FGF23 treatment alone, but in fact, PTH removed ETV1 from the locus (abundance of yellow, Fig. 5B). Globally, ChIP-seq tag density surrounding the ETV1 binding regions was examined (Fig. 5C). The 4,790 peak regions (Fig. 2D, Supplemental Table 2) were aligned and the sequencing density was examined in a 3 kb region surrounding each peak (±1.5 kb) in the vehicle, FGF23, and FGF23+PTH conditions. FGF23 increased the tag density compared to the Vehicle sample, however the addition of PTH to FGF23 caused a stark reduction of sequencing tag density indicating the vacating of ETV1 from the genome as observed around *Cyp24a1* (Fig. 5B). This is reflected in the overall cistrome, reducing ETV1 from 4,790 sites to 22 sites total (Fig. 5D), a remarkable reduction. These collective data verify that in response to FGF23, ETV1 was recruited and occupancy enriched on *Cyp24a1* within known enhancers which was completely reversed by PTH.

To test the cellular mechanisms directing reciprocal actions of PTH on FGF23-induced ETV1 protein localization *in vivo*, WT mice were injected as above with FGF23 or vehicle (3 h), +/− PTH (2 h, 230ng/g bw; Fig. 5E). Kidney nuclear protein demonstrated a decrease with the addition of PTH (Fig. 5F). We observed reciprocal, enhanced expression of *Cyp24a1* mRNA and suppressed expression of *Cyp27b1* mRNA with FGF23 (Fig. 5G). However, co-treatment with PTH significantly blunted these changes (Fig. 5G). These data are consistent with the idea that FGF23 and PTH have opposing actions on ETV1 function and supportive of ETV1’s role in controlling *Cyp24a1*.

The molecular ‘on/off’ switch acting between FGF23/1,25D and PTH to control *Cyp24a1* is unknown and we determined in our scRNAseq dataset from the FGF23-injected mice (see Fig. 1) that ubiquitination/proteosome degradation pathways were consistently upregulated in kidney (Fig. 5H). Some ETS TFs are controlled by protein turnover via interacting with the E3 ubiquitin ligase constitutive photomorphogenic-1 (mouse gene: *Cop1*; human gene: *RFWD2*), first characterized in plants as a rapid repressor of light-dependent photomorphogenesis transcription factors.^43,28,44–46^ Importantly, COP1 in mammals is inhibited by ERK/MAPK, consistent with FGF23/KL signaling. To test whether FGF23 controls kidney COP1 nuclear localization *in vivo*, WT mice were injected with FGF23 and COP1 expression tested by IF. At baseline, COP1 protein was detected primarily in megalin^+^ kidney cortex nuclei; however with FGF23 injection COP1 was detectable not only in the nucleus of the epithelial cells, but also dramatically localized to nephron cell cytoplasm (Fig. 5I; Fig. 5J, segmented COP1 signal with color-coded nuclear and cytoplasmic fractions), consistent with inactivation of COP1^47^.

To test for COP1 regulation of ETV1 as a novel FGF23-mediated mechanism of 1,25D control *in vitro*, ETV1-FLAG IP following FGF23 (50 ng/mL) and/or 1,25D (10 nM) treatments were performed in HEK-mKL cells (Fig. 5K). We found that ETV1 and COP1 could be co-precipitated with vehicle or 1,25D alone treatment, however in all lysates exposed to FGF23 (with or without 1,25D), ETV1-COP1 IPs were completely interrupted (Fig. 5K). These findings indicate that FGF23-mediated ETV1 expression may be controlled by direct COP1-mediated ubiquitination/degradation. The salt-inducible kinases (SIKs) are known to act downstream of PKA to block PTH actions on PT gene transcription, including the suppression of *Cyp24a1*.^14,48^ Thus, SIK inhibitors (SIKi) are ‘PTH mimetic’ and a useful reagent since parent HEK293 cells (and thus HEK-mKL cells) do not express the PTH receptor, PTH1R. To this end, HEK-mKL cells were treated with SIKi (HG-99101, 5 μM) for 4 h after 20 h of FGF23 (50 ng/mL), followed by immunoblot in the presence or absence of the general proteasome inhibitor MG132 (Fig. 5L). MG132 alone increased ETV1, supporting that cell ETV1 levels are controlled by proteasomal degradation (Fig. 5L). In the presence of MG132 and SIKi, ETV1 remained stabilized. However, with FGF23 and SIKi co-treatment, ETV1 was absent. When FGF23, SIKi, and MG132 were provided together, ETV1 was present (Fig. 5L), supporting that SIKi-mediated proteasomal degradation reduced ETV1. SIKi and MG132 had no effect on VDR, further suggesting independent control of this TF (Fig. 5L). We also tested ETV1 in the human PT cell line RPTEC, in which, versus control (Supplemental Fig. 6, duplicate lanes for each condition), proteasome inhibitor alone (MG132) increased ETV1, whereas SIKi (HG-99101)-mediated ETV1 degradation was rescued by co-treatment with MG132, confirming that protein turnover is a key regulatory step. The degradation of ETV1 downstream of SIKi can be rapid. Indeed, FGF23-mediated ETV1 production was completely reversed in HEK-mKL cells between 15–30 min (Supplemental Fig 7), supporting the concept that ETV1 is dynamically controlled to adapt to changing hormonal/mineral conditions.

Considering that FGF23 inhibited COP1-ETV1 interactions, to test whether SIKi mediated effects via COP1, an sh-COP1 strategy was used. In this regard, cells were treated with sh-COP1 or sh-EGFP control and treated with FGF23 (50 ng/mL) +/− SIKi (5 μM, 4 h). Treatment with FGF23 alone produced an increase in ETV1 that was ablated by SIKi (Fig. 5M) as above. However, in the absence of COP1, ETV1 degradation was significantly blocked (Fig. 5M) with no effect on VDR, demonstrating that COP1 was required for SIKi-mediated ETV1 turnover. Finally, in support of the suppressive actions of PTH on ETV1, we used our reporter constructs transfected into HEK-mKL cells (see Fig. 3N) and treated with SIKi (HG-99101) (5 μM) for 1 or 4 h after overnight treatment with 1,25D + FGF23 (Fig. 5N). At 4 h, the SIKi treated samples returned to 1,25D-only levels, corroborating the ETV1 specific actions at the C24 EBS that was mutated (Fig. 3N–P). Taken together, these results show that FGF23 and PTH reversibly control ETV1 binding to the genome, through mediating protein levels via reciprocal proteasomal regulation, underscoring targeted protein ubiquitination as a critical and rapid mediator of kidney FGF23-induced ETV1 expression.

In summary, our studies uncovered novel FGF23- and 1,25D-driven transcriptional reprogramming specifically localized to the renal PT via sensitive and coordinated interactions between ETV1 and VDR. We also found reciprocal actions of PTH on ETV1 to direct balanced control of *Cyp24a1*, an enzyme central to maintaining proper systemic circulating 1,25D concentrations. Further, targeting *Etv1 in vivo* showed that PT cell function was resistant to FGF23 resulting in elevated FGF23 and 1,25D altered systemic vitamin D metabolism. This critical system was found to be controlled by reversible interactions between the ETV1 and COP1-mediated degradation via the proteasome, providing new understanding and targets for the molecular control of 1,25D.

## Discussion

The control of the active form of vitamin D, 1,25D, is essential for maintaining a host of critical systemic processes including bone and mineral homeostasis, Ca^2+^ balance for muscle and nerve function, as well as immune processes. The ultimate synthesis steps in producing 1,25D occurs in select cells in the renal proximal tubule (PT) under the elegant control of FGF23, 1,25D and PTH. However, the molecular mechanisms regulating the balance of 1,25D synthesis at the genome level was unclear, thus impeding the ability to open new connections for strategies needed to address rare and common disorders related to these three hormones. Our Multiomic approach on kidney following temporal administration of FGF23 *in vivo* allowed the identification of the ETS family member ETV1 as PT-specific gene, which was detectable at baseline as being localized to the kidney PTS1/S2 with rapid induction at the mRNA and protein levels. This strategy allowed pinpointing unique transcriptional events due to FGF23, as previous bulk methodologies confirmed MAPK-responsive AP-1 TFs but did not identify PT-specific regulatory events.^37^ Further, since KL is localized to several nephron segments, clear resolution of FGF23 actions were now permissible. In support of our studies demonstrating ETV1’s critical functions in the control of 1,25D, previous data show that the TF ETS1, following overexpression, interacted with *Cyp24a1* promoter constructs *in vitro*, however the ETS TFs all share overlapping DNA binding domain motifs; thus it was not clear which ETS family member controlled 1,25D metabolism *in vivo*^49^. Additionally, data from vitamin D receptor (*Vdr*)-KO mice showed a reduction in total kidney *Etv1* mRNA^50^, but the ETV family has not been linked to date with normal or disordered FGF23/KL function, and our current results support novel, critical roles for ETV1 in mineral metabolism. Our results demonstrating blunted basal responses to FGF23 (reflected as a compensatory iFGF23 plasma increase), reduced *Cyp24a1* mRNA, and altered serum 25D vitamin D metabolite during conditional deletion of *Etv1* in kidney epithelial cells *in vivo* with no additional interventions, support that ETV1 is a critical component for FGF23-mediated control of *Cyp24a1* and vitamin D metabolism. Furthermore, when mice were challenged with a high-phosphate diet (elevating FGF23 levels), PTH increased in Cre^−^ mice, whereas Cre^+^ mice showed no significant increase, demonstrating an endocrine compensatory shift between regulators of vitamin D. We were unable to see a significant change in 1,25D; however, this is likely due to the known rapid and balanced actions of both FGF23 and PTH in response to the loss of *Etv1*. A strong indicator of vitamin D metabolic disruption was the elevation of 25(OH)D_3_ (25D) substrate, which occurs when the metabolic enzymes have reduced expression^5,9,51,52^. We did find that the 25D levels were elevated but not significantly (data not shown). Given the homeostatic nature of this system, the animal salvages normal 25D and 1,25D levels through hormonal changes and thus the minor elevation observed resulted in “normal” mineral and vitamin D levels. In the future, targeted *Cyp24a1* animal models may be necessary to exploit the EBS specifically and decouple the effects of *Cyp24a1* and *Cyp27b1* as we achieved in our prior enhancer deletion studies^9,10^ to help better clarify these effects.

Our data support a new paradigm where the actions of ETV1 and VDR are both physically and functionally intertwined. We found that ETV1-driven expression by CMV-promoter plasmids *in vitro* elevated *CYP24A1* mRNA without added 1,25D. This effect could be due to mass action of ETV1 on *CYP24A1* genomic regions, and reflected modest increases, similar to FGF23 administration alone in cultured HEK-mKL cells. *In vivo*, we found that not only do VDR and ETV1 co-localize on the exact regions in *Cyp24a1* that are known to be responsive to FGF23 and PTH via CRISPR-mediated deletion studies *in vivo*^9,11^, with FGF23 administration *in vitro*, these factors were more effectively co-precipitated in the presence of 1,25D. Further, *Cyp24a1* transcription was markedly elevated with combination treatment. These findings are consistent with our and others *in vivo* and *in vitro* data that FGF23 and 1,25D have independent effects on *Cyp24a1* and *Cyp27b1*, but in combination provide synergistic regulation of these enzymes, a response that is likely necessary for balanced, but potent control of 1,25D and thus Ca^2+^.^38^

The genomic mechanisms controlling *Cyp24a1* expression include a complex collection of enhancers with unique roles depending on the tissue. The kidney is one of the only tissues that has measurable levels of *Cyp24a1* expression by qPCR at baseline in a WT mouse on a standard diet. At homeostatic levels of 1,25D, FGF23, and PTH, the kidney drives the serum levels and general tissue availability for endocrine 1,25D to interact with the VDR. In most other tissues, *Cyp24a1* is below the limit of detection (by sensitive TaqMan qPCR), which helps maintain the available levels of 1,25D in said tissue through suppression of 1,25D catabolism. Outside of a nutritional deficiency of Ca/P, disease, or development, a healthy individual will rarely increase the 1,25D levels enough to elicit *Cyp24a1* expression in non-kidney tissues. As mentioned, we identified that the PT-specific DS1 region was important for the regulation of *Cyp24a1* by both FGF23 and PTH and the more ubiquitous DS2 enhancer controlled the 1,25D response in our prior genomic deletions of enhancers for *Cyp24a1*.^10^ Additionally, deletion of DS1 reduced the baseline of *Cyp24a1*, but deletion of either the DS2 or the *Cyp24a1* promoter VDREs (C24-V1V2 mice) did not reduce the baseline.^10,38^ Thus, we previously determined the DS1 region must be vital for the enhanced baseline expression of *Cyp24a1* and that PTH and FGF23 control these activities as deletion of the DS1 greatly diminished the response to both FGF23 and PTH, as well.^10^ Here, we found that ETV1 was present at baseline across the DS1 region as well as the promoter and part of the DS2 region. This binding was enhanced by the treatment of FGF23 after 1 h (Fig. 2A) with what we believe was most likely due to increasing ETV1 protein at 1 h (Fig. 1H), although we have not ruled out an activating modification to ETV1 itself, as ETV1 is known to be phosphorylated by PKA and acetylated by p300.^35,53^

We recently described the rapid actions of PTH and SIK inhibitors through CREB and coactivators like CRTC2 and CBP for both *Cyp27b1* and *Cyp24a1*.^14,48^ CREB along with CRTC2 and CBP were recruited and had enhanced genomic occupancy at *Cyp27b1* however, CREB and coactivators were not reduced at *Cyp24a1* in response to PTH or the SIK inhibitors. In Fig. 5B, we found a remarkable loss of ETV1 in the presence of PTH alone and PTH was even potent enough to not only reverse the ETV1 mediated occupancy increases by FGF23, but it further excluded ETV1 completely from the genome, reducing the cistrome of ETV1 from 4,790 sites to only 22 (Fig. 5D). This incredible exclusion of ETV1 by PTH demonstrates that ETV1, not CREB, was likely responsible for the PTH mediated suppression. These data also offer a clear distinction of how a single treatment, PTH, can have opposite activities on these genes, inducing *Cyp27b1* through CREB and at the same time, excluding ETV1 from the enhancers of *Cyp24a1* and thus reducing its activity. Certainly, the 1,25D-dependent enhancement of ETV1-VDR immunoprecipitation, taken together with our data showing co-localized binding of ETV1/VDR within *Cyp24a1 in vivo* by ChIP assay and *in vitro* by gene expression and ChIP, supports that FGF23 and 1,25D, acting through ETVs and VDR, respectively are required for high-potency control of 1,25D synthesis. Further, ETV1 sits at the nexus between FGF23 activation and PTH suppression which is required for the rapid, high-capacity regulation of Ca^2+^ and thus essential for normal metabolic processes and life. Our data in the *Etv1* deleted mice showed less impact on kidney *Cyp27b1* mRNA expression. Since *Cyp27b1* is the direct rate-limiting enzyme for 1,25D synthesis, it would not be surprising that the mechanisms controlling the context-dependent production of 1,25D by *Cyp27b1* through interactions between FGF23, PTH, and 1,25D may be influenced by multiple regulatory pathways and more complex genomic interactions. Indeed, *Cyp27b1* has no promoter-proximal enhancer, whereas *Cyp24a1* is governed by a canonical promoter with multiple enhancer elements, potentially contributing to their distinct transcriptional control of these enzymes within the same cell.

**Figure 6:**
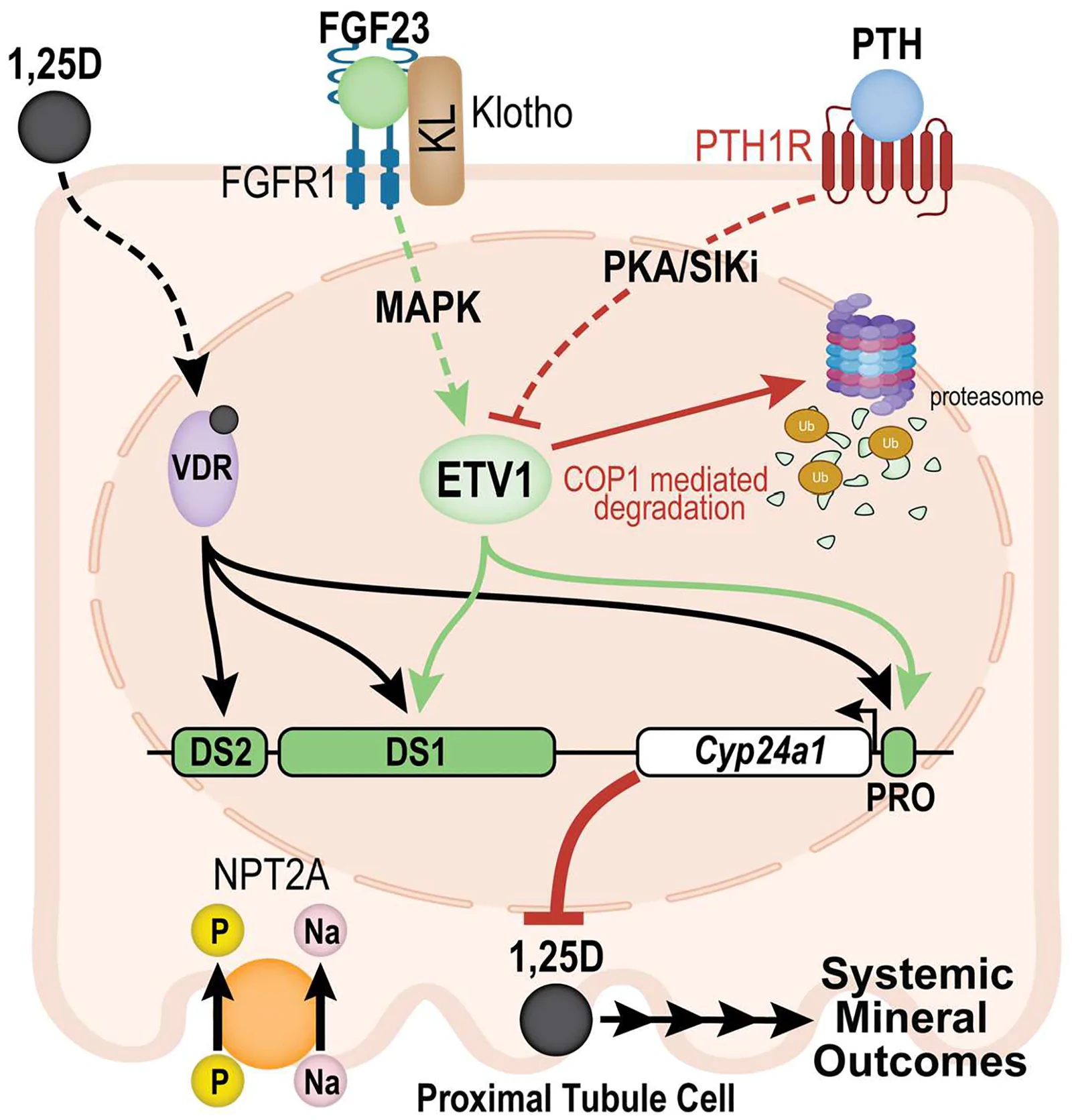
Schematic summary of FGF23 control over *Cyp24a1* genomic expression. ETV1 is directed by FGF23 signaling to bind to the *Cyp24a1* downstream 1 (DS1) enhancer and promoter (PRO) regions in the kidney. 1,25D directs VDR binding to the downstream 2 (DS2), DS1, and PRO regions where it interacts with ETV1 in the control of *Cyp24a1*. ETV1 levels are controlled by a COP1-mediated degradation after protein kinase A (PKA) through PTH, or salt-inducible kinase (SIK) inhibition (SIKi). The outcomes of these activities control the circulating levels of vitamin D metabolites like 1,25D and ultimately systemic mineral outcomes.

The ETV protein stability can be influenced by several mechanisms, the most important of which may be through COP1-mediated ubiquitination and proteasome degradation.^54^ COP1 was originally described as a key transcriptional regulator in plants, in which light increases nuclear export of COP1, reducing degradation of TFs responsible for photomorphogenesis.^43^ In higher organisms, COP1 nuclear export is inhibited by ERK/MAPK to permit ETV activity, consistent with known FGF23/KL/FGFR signaling cascades.^46^ The dichotomy of FGF23/MAPK and PTH/SIK on COP1-mediated degradation of ETV1 and ETV1 DNA binding has not been tested in the kidney. We demonstrated that in the presence of FGF23 COP1-ETV1 interactions are inhibited *in vitro* and COP1 becomes localized to the proximal tubule cytoplasm *in vivo*, consistent with findings that proteasome-mediated ETV1 degradation was inhibited with shRNA specifically targeting COP1. COP1 has regulatory roles in other critical endocrine systems. One notable example is the control of insulin release, where it was shown that deletion of ETVs from pancreas reversed COP1-sensitve glucose induction by restoring insulin secretion.^28^ We demonstrated that ETV1-COP1 co-immunoprecipitation was lost in response to FGF23 treatment (and with FGF23+1,25D), which would then permit ETV1 transcriptional function. In contrast, treatment with SIKi, known to be PTH-mimetic^14,55^ prior to, and concurrent with, FGF23 treatment resulted in loss of mature ETV1 protein, but not VDR. These findings underscore that the COP1-ETV regulatory system cannot only inhibit ETVs prior to production, but also post-translation, which would be essential for a reversible system that was sensitive to activation by FGF23/1,25D and then inhibition by PTH, or vice-versa. Further, the observation that VDR was still present after SIKi treatment in the absence of ETV1 supports the concept that although FGF23 and 1,25D share genomic targets, their ‘corresponding’ TFs, ETV1 and VDR, respectively, can be individually regulated. This new mechanism for FGF23 control of *Cyp24a1* expression is summarized in Fig. 6.

In summary, we demonstrated herein that ETV1, expressed almost exclusively in the kidney PT in response to FGF23, binds VDR in the presence of 1,25D, and within critical regulatory elements in the *Cyp24a1* promoter and enhancer regions. In contrast, PTH signaling promotes COP1-dependent proteasomal turnover of ETV1, rapidly dismantling this transcriptional complex and providing a mechanistic basis for the counter-regulatory endocrine control of renal vitamin D metabolism.

## Material and Methods

### Research animals

Animal studies were approved by, and performed, according to the Institutional Animal Care and Use Committee (IACUC) of the Indiana University School of Medicine and the University of Wisconsin-Madison, and complied with the NIH guidelines for the use of animals in research.

#### Hormone administration:

C57BL/6J male and female mice (9-weeks-old) were purchased from the Jackson Laboratory and housed for 1 week. At 10-weeks of age, mice were treated with a single intraperitoneal injection of 250 ng/g body weight of recombinant human FGF23 (R&D Systems, Cat no. 2604-FG-025/CF) and euthanized at baseline (0h), or after 1, 4, 12 h. For nuclear isolation preparations, C57Bl6 mice (n=5) were injected with vehicle (1X PBS) or FGF23 (250 ng/g bw) for (3 h) then either PTH (230 ng/g bw) or vehicle for 2 additional hours. Mice were euthanized by CO_2_ inhalation/cervical dislocation, and blood was collected by cardiac puncture for serum and plasma (collected in EDTA tubes). For scRNAseq experiments, male-female kidney pairs were digested, and cells were isolated as previously described.^18^ Total kidney RNA from *Fgf23*- and *KL*-KO mice^15,56^ at 5 w of age were used for qPCR.

#### Doxycycline-inducible ETV1^fl/fl^/Pax8-Cre mice:

Kidney epithelial cell-specific deletion of *Etv1* was obtained via breeding the flox-*Etv1* allele (from Dr. David Park, NYU;^57–59^) onto the doxycycline (DOX)-inducible *Pax8*-rtTA/LC1-Cre (herein referred to as ‘*Pax8*-Cre’)^42^, obtained from Dr. Nirupama Ramkumar (Univ. of Utah Nephrology); 6–7 week-old Cre^+/−^ mice were placed on provided DOX treatment (2 mg/mL) for 12 days followed by a 4 w recovery period (normal water), and placed on a normal phosphate (0.6% P, 1% Ca) or a high phosphate (2% P, 1% Ca) diet the last 2 w of the recovery period prior to euthanasia. Littermates were used as controls in all experiments.

### Single cell 10X Multiome library preparation and data processing

For scMultiome, male and female kidneys were digested, and cells were isolated as previously described^60^. In brief, mouse kidney was minced and homogenized in Nuclei EZ lysis buffer following a filtration through a 40- μm cell strainer and centrifuged for 5 min at 500 g. For each sequencing, 10,000 cell recovery was targeted and applied a single cell master mix with lysis buffer and reverse transcription reagents, according to the Chromium Single Cell 3’ Reagent Kits V3 User Guide, CG000183 Rev A (10X Genomics, Inc.). This was followed by cDNA synthesis and library preparation. Nuclei partitioning, library preparation, and sequencing were performed following the 10X Chromium protocol. All libraries were sequenced on the Illumina NovaSeq6000 platform in paired-end mode (28bp + 91bp). Cell Ranger-ARC 2.0.2 (http://support.10xgenomics.com/) was utilized to process the raw sequence data generated. The 10x Genomics multiome RNA + ATAC data were processed using Seurat^61^ and Signac^62^. RNA-seq data were processed following the standard workflow. Low-quality droplets were defined as those containing <1000 total unique molecular identifiers, or the percentage of mitochondrial reads was >10%. scDblFinder^63^ was used to identify doublet. Low-quality droplets and doublets were removed. We used SCTransform to normalize RNA counts. Samples were integrated using FindIntegrationAnchors in Seurat package.^61^ After scaling the integrated data, the first 30 Principal components from Principal component analysis (PCA) were used to cluster the cells by a shared nearest neighbor (SNN) modularity optimization-based clustering algorithm visualization of the resulting clusters were performed with Seurat package. ATAC-seq peaks were identified for each sample separately using MACS2.^64^ The peaks from different samples are merged to form a union peak. TSSEnrichment and NucleosomeSignal functions were used to compute the per-cell quality. Cells with nucleosome signal score >2 and TSS enrichment score <1.5 were removed. Term Frequency Inverse Document Frequency (TF-IDF) was used to normalize ATAC peaks and LSI to reduce the dimensionality of ATAC data. FindIntegrationAnchors was used to integrate ATAC-seq data. Finally, 1–50 PCA dimensions and 2–50 LSI dimensions were used to construct the weighted nearest neighbor (WNN) graph for clustering. Clusters were annotated to cell types using SingleR package^65^, using label transfer from known scRNA-seq^66^ as reference, respectively as well as marker genes. Differential gene expression analysis was analyzed using Wilcoxon Rank Sum test with Seurat package. Gene ontology and KEGG pathway functional analysis was performed on differential expression gene with false discovery rate cut-off of 0.05 and absolute value of log2 of fold change cut-off of 0 using DAVID.^67,68^

### RNAseq sex hashing

In addition to the female X chromosome *Xist* gene and male Y chromosome *Kdm5d* and *Eif2s3y* genes that were used as markers to distinguish male and female cells within the pooled dataset, additional filters were applied to separate male and female cells in PT^22^. Specifically, in PT-S1, male cells were identified with markers *Slc22a28*^*high*^, *Cyp2e1*^−^ and *Xist*^−^ whereas female cells were *Spp2*^*high*^, *Cyp2d26*^*high*^, *Cyp4a14*^*−*^ and *Xist*^+^. Male PT-S2 cells were identified with *Cyp2e1*^*high*^, *Nat8*^*high*^ and *Xist*^*−*^ whereas female S2 cells were predominantly *Cyp4a14*^*high*^ and *Xist*^+^. The cells from PT-S3 were identified as male with *Cyp7b1*^+^, *Slc7a13*^*high*^ and *Xist*^*−*^ whereas *Slc7a12*^+^ and *Xist*^+^ were identified as female. Analysis of sex dependent FGF23 bioactivity was performed using a false discovery rate (FDR) less than 5%.

### Serum biochemical parameters

Serum Ca^2+^, P, alkaline phosphatase, BUN, and creatinine were measured by automation (COBAS) as previously described.^15,69^ Plasma FGF23 was tested using rodent-specific commercial ELISAs for bioactive, intact FGF23 (‘iFGF23’). 1,25D and derivatives were assessed by LC-MS/MS as previously reported.^38,52^

### Kidney RNA isolation and qPCR

Kidneys were harvested and homogenized in 1 ml of TRIzol reagent (15596018; Thermo Fisher Scientific; Waltham, MA) according to the manufacturer’s protocol using a Bullet Blender (Next Advance, Inc.), then further purified using the RNeasy Kit (74104; Qiagen, Germantown, MD). RNA samples described in Results were tested for specific gene expression with intron-spanning primers, as well as mouse and human *β-actin* or *Gapdh* as internal controls for RT-qPCR (see Supplemental Table 3). All qPCR primers were purchased as pre-optimized reagents (Applied Biosystems/Life Technologies, Inc.; Waltham, MA) and the TaqMan One-Step RT-PCR kit was used to perform qPCR. PCR conditions for all experiments were 30 minutes 48°C, 10 minutes 95°C, followed by 40 cycles of 15 seconds 95°C and 1 minute 60°C. The data were collected and analyzed by a StepOne Plus system (Applied Biosystems/Life Technologies, Inc.). The expression levels of mRNAs were calculated relative to appropriate controls, and the 2^−ΔΔCT^ method described by Livak was used to analyze the data^45^.

### ChIP followed by Sequencing (ChIP-seq)

Chromatin Immunoprecipitation (ChIP) was performed using the ETV1 antibody (Invitrogen PA5–77975)^70^. VDR data were previously published^14,38^. ChIP on the mouse kidney nephron was performed as described previously in triplicate (male mice, n=3 per treatment) with several modifications^9,10,14^. The isolated DNA (or Input DNA acquired prior to precipitation) was then validated by quantitative real time PCR (qPCR) and further prepared for ChIP-seq analysis. ChIP-seq libraries were prepared as previously described^71,72^ with the following exceptions: ChIP-seq libraries were prepared using the NEBNext Ultra II DNA kit (NEB, #E7645S) with the NEBNext Multiplex Oligos for Illumina (NEB, #E6440S) according to the manufacturer’s protocols with library concentrations measured by Qubit (Life Technologies) and library size distribution by Bioanalyzer 2100 (Agilent). Libraries were sequenced on a NovaSeqX (Azenta/Genewiz, Waltham, MA). Paired end, 150 bp sequencing with a target of 30+ million reads was performed. ChIP-sequencing files were processed by HOMER^73^ and MACS3^74^ for ChIP peaks (as in Supplemental Table 2) as well as EdgeR^75^, and DESeq2^76^ for replicate fold change (as in Supplemental Table 1). The raw and processed data have been deposited in the GEO archives (mm9 genome, see Data availability). The displayed data tracks are from bigWig file replicates that were averaged using wiggletools mean function. All replicate averaged ChIP data were normalized to 10 million reads and to Input DNA for display in the UCSC Genome Browser as overlaid track hubs.

### *In vitro* cell model studies

HEK293 cells stably transfected with membrane bound-KL (HEK-mKL cells;^77^ were cultured in Alpha MEM (Hyclone) supplemented with 10% fetal bovine serum, 1% L-Glutamine 100X Solution (Hyclone), 50,000U Penicillin-Streptomycin Solution (Hyclone) at 37°C under 5% CO_2_ conditions. The in vitro experimental conditions were performed as follows: 2.5 × 10^5^ cells were plated on 24-well plates (Midwest Scientific) overnight and treated with PBS or recombinant human FGF23 for times and doses described in Results and figure legends. Cells were treated pre-treated with proteasome inhibitor (MG-132, 5 μM; Cell Signaling Technology, Cat no. 2194S) and sequentially treated with FGF23 and then SIK inhibitor (HG-99101, 5 μM; Medchem Express). Renal proximal tubule epithelial cell line (RPTEC-TERT) was obtained from ATCC (Cat no. CRL-4031). Cells were cultured in DMEM-F12 (ATCC) and supplemented with hTERT immortalized RPTEC growth kit (ATCC). Protein cell lysates were collected with 200 μl 1X cell lysis buffer (Cell Signaling) with 0.1mM 4-(2-Aminoethyl) benzenesulfonyl fluoride hydrochloride (AEBSF, Santa Cruz). Cell RNA was extracted using ISOLATE II RNA mini kit (Bioline), according to the manufacturer’s directions, followed by qPCR analysis as described above.

### Plasmids and transfections

CMV-ETV1–3XFLAG (pRP[Exp]-EGFP/Puro-CMV>hETV1[NM_001370555.1](co)*/3xFLAG) and CMV-ETV5–3xFLAG were designed with VectorBuilder software using optimized human codons and assembled by the same company. FuGENE6 (Promega) transfection reagent was used with 2 μg of plasmid in a 2:1 ratio according to the manufacturer’s instructions, and administered to either 1×10^6^ HEK-mKL cells for 48 h prior to treatment with FGF23 (50 ng/mL) +/− 1,25D (10 nM) for the times listed in Results and in Figure legends. For RPTEC cells, TransfeX transfection reagent (ATCC) was used with CMV-ETV1–3XFLAG cDNA plasmid 2μg of plasmid 1:1 ratio according to the manufacturer’s instructions for 48 h.

### Luciferase Reporter Assays

Genomic sequence (wildtype and mutant) of the mouse *Cyp24a1* promoter sequence from +60 to −74 bp surrounding the transcriptional start site were synthesized as gBlocks (IDT, Coralville, IA). ETS binding site (EBS, −136/−147) WT 5’-CATCCTCTTTC was mutated to 5’-CgggaaaTTTC. gBlock fragments were cloned into the pGL3-basic-luciferase reporter plasmid using Gibson Assembly^78^ and verified by restriction digest and Sanger sequencing. HEK-mKL cells were plated at a density of 1×10^5^ cells per well of a 24-well plate prior to transfection. Reporter plasmids (250 ng/well) and/or CMV-ETV1–3xFLAG (50 ng/well) were transfected with the Lipofectamine 3000 (Invitrogen) reagent following manufacturer’s recommendations. Wells were treated with vehicle (Veh, EtOH/PBS), 10 nM 1,25(OH)_2_D_3_, 50 ng/mL human FGF23, or 1,25D + FGF23 for 24 h in triplicate. Additionally, cells were treated with SIK inhibitor (HG-99101, 5 μM) for 1 or 4 h with or without 50 ng/mL human FGF23 in triplicate. All assays are representative of at least 3 repeat assays.

### Immunoprecipitations

To perform immunoprecipitations, HEK-mKL cells were transfected with the CMV-ETV1–3xFLAG cDNA construct(s) as above, then lysed with 10X Lysate buffer + AEBSF as described. Pierce^™^ Anti-DYKDDDDK Magnetic Agarose (100 μL; Thermo Fisher Scientific) was added to the lysates and incubated overnight at 4°C with rocking. Lysates were centrifuged at 13,000 xg and washed 3 times in 1X PBS; 50 μg of total protein was electrophoresed for immunoblots with the concentrations of specific primary antibodies described above.

### Lentiviral shRNA vectors

Lentiviruses carrying templates for ETV1sh-EGFP-PuroU6 (pLV[shRNA]-EGFP:T2A:Puro-U6>hETV1[shRNA]), COP1-mCherry-PuroU6 (pLV[shRNA]-mCherry:T2A:Puro-U6>hCOP1) and Scramblesh-EGFP-PuroU6 (pLV[shRNA]-EGFP/Puro-U6>Scramble_shRNA) control shRNAs were designed using VectorBuilder design software (Vectorbuilder.com), then assembled and amplified by the same company. In brief, 1×10^6^ HEK-mKL cells were transduced with were transduced with 10 MOI for 5 days and assayed for mRNA or protein after treating with FGF23 (50 ng/mL) +/− 1,25D (10 nM) for the times listed in Results and Figure legends.

### Immunoblotting

HEK-mKL cells were lysed with 300 μL 1X Lysis buffer (Cell Signaling Technology, Inc., Danvers, MA, USA; 9803-S) with 1 μg/mL 4-(2-aminoethyl) benzenesulfonyl fluoride hydrochloride protease inhibitor (AEBSF; SBR00015, Sigma-Aldrich, Inc.;) as previously performed.^20^ Total cell lysate protein concentrations were determined with the Better Bradford Kit (23236; Thermo-Fisher Scientific) according to the manufacturer s instructions. Immunoblot analyses were performed with 50–100 μg of cellular lysates. The blots were incubated with 1:1000 primary antibody to ETV1 (Cat. no. ab314874; Abcam), COP1 (Cat. no. ab70889; Abcam), or VDR (Cat. no. 12550S; Cell Signaling Technology) overnight at 4°C then incubated with secondary antibody at 1:2000 (anti-rabbit horseradish peroxidase [HRP]; Cell Signaling Technol.) for 2 h at room temperature. Blots were stripped using SDS-glycine and reprobed with 1:10,000 anti-β-actin-HRP (A3854; Sigma-Aldrich). Detection was performed using the ECL Prime Western Blotting Detection Reagents (Amersham-GE Healthcare, Pittsburgh, PA, USA) and a GE AB1600 digital imager.

### Immunofluorescence and imaging

Kidneys from 1X PBS, FGF23 (250 ng/g bw), and/or PTH (230 ng/g bw) treated mice (10-weeks of age, female; 3–5 h) were removed and fixed in 4% PFA overnight, then stored in 70% EtOH until paraffin embedding. Paraffin-embedded kidney sections (10 μm) were stained using anti-megalin primary antibody (1:1000; sc-515750 FITC, Santa Cruz), anti-ETV1 (1:50; ab314874; Abcam), and anti-COP1 (1:200; ab70889; Abcam) followed by a treatment with the polymer detection kit ImmPRESS^®^ HRP Horse Anti-Rabbit IgG, Peroxidase (MP-7401; Vector Labs); subsequently the signal amplification kit TSA TMR reagent pack (Akoya Biosciences) was applied. A negative control without primary antibody was used to ensure the absence of nonspecific binding of secondary antibodies. Single-plane tile images of the kidney were collected at the ICBM Imaging Facility (Indianapolis, IN) using a Leica TCS SP8 confocal/two-photon microscope (Leica Microsystems, Inc., Buffalo Grove, IL) equipped with a Leica HC PL APO CS2 63x/1.3 Gly objective lens. Sequential laser scanning was performed as follows: Seq1 – PMT1 (DAPI) at 405 nm excitation / 415–483 nm emission and PMT3 (endogenous fluorescence) at 638 nm excitation / 650–750 nm emission; Seq2 – PMT2 (COP1) at 488 nm excitation / 500–545 nm emission; Seq3 – PMT3 (Megalin) at 552 nm excitation / 560–630 nm emission. Images were acquired at 12-bit depth with a resolution of 1024 × 1024 pixels (0.172 μm/pixel), using bidirectional X-scanning at a speed of 400 Hz, a pinhole set to 1 AU, and a line averaging of 2. The hardware scanning configuration was maintained consistently across all samples. Image stitching was performed using Leica LAS X software (version 3.5.7). Images were analyzed using Imaris software (v10.2; Oxford Instruments), available at the Indiana Center for Biological Microscopy (Indianapolis, IN). The ‘Surfaces’ module was used to segment COP1 and nuclei. Saturated COP1 signal artifacts observed in the vasculature were filtered out during segmentation by referencing erythrocyte outlines in the autofluorescence channel. COP1 signal was further classified based on its spatial relationship to nuclei using the machine learning classification module, eliminating any remaining artifacts, primarily those present in the tubular lumen. COP1 localized to nuclei was color-coded gray, while cytoplasmic COP1 was color-coded orange.

### Quantification and statistical analysis

Updated R software packages with robust affiliated statistics were used to analyze the 10X Multiome datasets and GraphPad Prism version 10.6.1. Statistical analyses of the *in vitro* data were performed by one-way ANOVA to assess the differences between the same cell line responses to recombinant proteins, chemical inhibitors, and cDNA transfections. High phosphate diet results were obtained using Two-way ANOVA (genotype x diet). Genotype (Cre−/Cre+) Statistical analyses of the *in vivo* data were performed by Welch’s T-test. Two-way ANOVA to assess differences between treatments and genotypes. There were no differences between sexes; thus, groups were analyzed together. Means and standard deviations were used in bar graphs and kinetic curves. Significant changes were considered at *P* < 0.05.

## Supplementary Material

1

## Data Availability

The Multiome scRNAseq/scATACseq data is deposited in the NCBI’s Gene Expression Omnibus (GEO) database GSE337338. The ETV1 ChIP-seq from mouse kidney is deposited in GEO GSE308142 with additional VDR data in GSE275331 and Kidney Input samples from GSM3898546.

## References

[1] Olauson H., Mencke R., Hillebrands J. L. & Larsson T. E. Tissue expression and source of circulating αKlotho. Bone 100, 19–35 (2017). 10.1016/j.bone.2017.03.043 28323144

[2] Andrukhova O. FGF23 promotes renal calcium reabsorption through the TRPV5 channel. The EMBO Journal, n/a–n/a (2014). 10.1002/embj.201284188 PMC3983685 24434184

[3] Ramnitz M. S. Phenotypic and Genotypic Characterization and Treatment of a Cohort With Familial Tumoral Calcinosis/Hyperostosis-Hyperphosphatemia Syndrome. J Bone Miner Res 31, 1845–1854 (2016). 10.1002/jbmr.2870 27164190 PMC5071128

[4] Autosomal dominant hypophosphataemic rickets is associated with mutations in FGF23. Nat Genet 26, 345–348 (2000). 10.1038/81664 11062477

[5] Meyer M. B. & Pike J. W. Genomic mechanisms controlling renal vitamin D metabolism. J Steroid Biochem Mol Biol 228, 106252 (2023). 10.1016/j.jsbmb.2023.106252 36657729 PMC10006327

[6] Ide N. In vivo evidence for a limited role of proximal tubular Klotho in renal phosphate handling. Kidney Int 90, 348–362 (2016). 10.1016/j.kint.2016.04.009 27292223

[7] Olauson H. Targeted deletion of Klotho in kidney distal tubule disrupts mineral metabolism. J Am Soc Nephrol 23, 1641–1651 (2012). 10.1681/ASN.2012010048 22878961 PMC3458458

[8] Meyer M. B. & Pike J. W. Mechanistic homeostasis of vitamin D metabolism in the kidney through reciprocal modulation of Cyp27b1 and Cyp24a1 expression. J Steroid Biochem Mol Biol 196, 105500 (2020). 10.1016/j.jsbmb.2019.105500 31629064 PMC6954286

[9] Meyer M. B. Targeted genomic deletions identify diverse enhancer functions and generate a kidney-specific, endocrine-deficient Cyp27b1 pseudo-null mouse. J Biol Chem 294, 9518–9535 (2019). 10.1074/jbc.RA119.008760 31053643 PMC6579472

[10] Meyer M. B. A chromatin-based mechanism controls differential regulation of the cytochrome P450 gene *Cyp24a1* in renal and nonrenal tissues. J Biol Chem 294, 14467–14481 (2019). 10.1074/jbc.RA119.010173 31439663 PMC6768633

[11] Meyer M. B. A kidney-specific genetic control module in mice governs endocrine regulation of the cytochrome P450 gene *Cyp27b1* essential for vitamin D_3_ activation. J Biol Chem 292, 17541–17558 (2017). 10.1074/jbc.M117.806901 28808057 PMC5655528

[12] Meyer M. B., Goetsch P. D. & Pike J. W. A downstream intergenic cluster of regulatory enhancers contributes to the induction of *CYP24A1* expression by 1alpha,25-dihydroxyvitamin D_3_. J Biol Chem 285, 15599–15610 (2010). 10.1074/jbc.M110.119958 20236932 PMC2865326

[13] Pike J. W. & Meyer M. B. The unsettled science of nonrenal calcitriol production and its clinical relevance. J Clin Invest 130, 4519–4521 (2020). 10.1172/JCI141334 32716362 PMC7456237

[14] Meyer M. B. Rapid genomic changes by mineralotropic hormones and kinase SIK inhibition drive coordinated renal Cyp27b1 and Cyp24a1 expression via CREB modules. J Biol Chem, 102559 (2022). 10.1016/j.jbc.2022.102559 36183832 PMC9637672

[15] Clinkenbeard E. L. Increased FGF23 protects against detrimental cardio-renal consequences during elevated blood phosphate in CKD. JCI Insight 4 (2019). 10.1172/jci.insight.123817 PMC6478421 30830862

[16] Hasegawa H. Direct evidence for a causative role of FGF23 in the abnormal renal phosphate handling and vitamin D metabolism in rats with early-stage chronic kidney disease. Kidney Int 78, 975–980 (2010). 10.1038/ki.2010.313 20844473

[17] Hu M. C. Klotho deficiency causes vascular calcification in chronic kidney disease. J Am Soc Nephrol 22, 124–136 (2011). 10.1681/asn.2009121311 21115613 PMC3014041

[18] Urakawa I. Klotho converts canonical FGF receptor into a specific receptor for FGF23. Nature 444, 770–774 (2006). 10.1038/nature05315 17086194

[19] Portale A. A. Characterization of FGF23-Dependent Egr-1 Cistrome in the Mouse Renal Proximal Tubule. PLOS ONE 10, e0142924 (2015). 10.1371/journal.pone.0142924 26588476 PMC4654537

[20] Nutchey B. K. Molecular action of 1,25-dihydroxyvitamin D3 and phorbol ester on the activation of the rat cytochrome P450C24 (CYP24) promoter: role of MAP kinase activities and identification of an important transcription factor binding site. Biochem J 389, 753–762 (2005). 10.1042/BJ20041947 15836435 PMC1180726

[21] Dwivedi P. P. Role of MAP kinases in the 1,25-dihydroxyvitamin D3-induced transactivation of the rat cytochrome P450C24 (CYP24) promoter. Specific functions for ERK1/ERK2 and ERK5. J Biol Chem 277, 29643–29653 (2002). https://doi.org/M204561200 10.1074/jbc.M204561200 12048211

[22] Dwivedi P., Omdahl J., Kola I., Hume D. & May B. Regulation of rat cytochrome P450C24 (CYP24) gene expression. Evidence for functional cooperation of Ras-activated Ets transcription factors with the vitamin D receptor in 1,25-dihydroxyvitamin D(3)-mediated induction. J Biol Chem 275, 47–55 (2000). 10617584 10.1074/jbc.275.1.47

[23] Lu B. C. Etv4 and Etv5 are required downstream of GDNF and Ret for kidney branching morphogenesis. Nat Genet 41, 1295–1302 (2009). 10.1038/ng.476 19898483 PMC2787691

[24] Yambire K. F. Mitochondrial biogenesis is transcriptionally repressed in lysosomal lipid storage diseases. eLife 8 (2019). 10.7554/elife.39598 PMC6379092 30775969

[25] Bi H., Zhang M., Wang J. & Long G. The mRNA landscape profiling reveals potential biomarkers associated with acute kidney injury AKI after kidney transplantation. PeerJ 8, e10441 (2020). 10.7717/peerj.10441 33312771 PMC7703406

[26] Qi T. Function and regulation of the PEA3 subfamily of ETS transcription factors in cancer. Am J Cancer Res 10, 3083–3105 (2020). 33163259 PMC7642666

[27] Kandemir B., Gulfidan G., Arga K. Y., Yilmaz B. & Kurnaz I. A. Transcriptomic profile of Pea3 family members reveal regulatory codes for axon outgrowth and neuronal connection specificity. Scientific Reports 10, 18162 (2020). 10.1038/s41598-020-75089-3 33097800 PMC7584614

[28] Suriben R. β-Cell Insulin Secretion Requires the Ubiquitin Ligase COP1. Cell 163, 1457–1467 (2015). 10.1016/j.cell.2015.10.076 26627735

[29] Agoro R. Dynamic Single Cell Transcriptomics Defines Kidney FGF23/KL Bioactivity and Novel Segment-Specific Inflammatory Targets. bioRxiv, 2024.2005.2024.595014 (2024). 10.1101/2024.05.24.595014 PMC11928261 39828039

[30] Ransick A. Single-Cell Profiling Reveals Sex, Lineage, and Regional Diversity in the Mouse Kidney. Dev Cell 51, 399–413 e397 (2019). 10.1016/j.devcel.2019.10.005 31689386 PMC6948019

[31] Vinas J. L. Sex diversity in proximal tubule and endothelial gene expression in mice with ischemic acute kidney injury. Clin Sci (Lond) 134, 1887–1909 (2020). 10.1042/CS20200168 32662516

[32] Zhang Z., Verheyden J. M., Hassell J. A. & Sun X. FGF-Regulated Etv Genes Are Essential for Repressing Shh Expression in Mouse Limb Buds. Developmental Cell 16, 607–613 (2009). 10.1016/j.devcel.2009.02.008 19386269 PMC3541528

[33] Wei G. H. Genome-wide analysis of ETS-family DNA-binding in vitro and in vivo. EMBO J 29, 2147–2160 (2010). 10.1038/emboj.2010.106 20517297 PMC2905244

[34] Oh S., Shin S. & Janknecht R. ETV1, 4 and 5: An oncogenic subfamily of ETS transcription factors. Biochimica et Biophysica Acta (BBA) - Reviews on Cancer 1826, 1–12 (2012). 10.1016/j.bbcan.2012.02.002 22425584 PMC3362686

[35] Cooper C. D., Newman J. A., Aitkenhead H., Allerston C. K. & Gileadi O. Structures of the Ets Protein DNA-binding Domains of Transcription Factors Etv1, Etv4, Etv5, and Fev: DETERMINANTS OF DNA BINDING AND REDOX REGULATION BY DISULFIDE BOND FORMATION. J Biol Chem 290, 13692–13709 (2015). 10.1074/jbc.M115.646737 25866208 PMC4447949

[36] Garg A. Etv transcription factors functionally diverge from their upstream FGF signaling in lens development. eLife 9 (2020). 10.7554/elife.51915 PMC7069720 32043969

[37] Agoro R. Dynamic single cell transcriptomics defines kidney FGF23/KL bioactivity and novel segment-specific inflammatory targets. Kidney Int 107, 687–699 (2025). 10.1016/j.kint.2024.12.014 39828039 PMC11928261

[38] Meyer M. B. In vivo contribution of Cyp24a1 promoter vitamin D response elements. Endocrinology (2024). 10.1210/endocr/bqae134 PMC11487884 39363152

[39] Suzuki T. A novel scaffold protein, TANC, possibly a rat homolog of Drosophila rolling pebbles (rols), forms a multiprotein complex with various postsynaptic density proteins. Eur J Neurosci 21, 339–350 (2005). 10.1111/j.1460-9568.2005.03856.x 15673434

[40] Farrow E. G., Davis S. I., Summers L. J. & White K. E. Initial FGF23-mediated signaling occurs in the distal convoluted tubule. J Am Soc Nephrol 20, 955–960 (2009). 10.1681/ASN.2008070783 19357251 PMC3060419

[41] Patel T. D. Peripheral NT3 Signaling Is Required for ETS Protein Expression and Central Patterning of Proprioceptive Sensory Afferents. Neuron 38, 403–416 (2003). 10.1016/s0896-6273(03)00261-7 12741988

[42] Traykova-Brauch M. An efficient and versatile system for acute and chronic modulation of renal tubular function in transgenic mice. Nat Med 14, 979–984 (2008). 10.1038/nm.1865 18724376 PMC3446847

[43] Torii K. U., McNellis T. W. & Deng X. W. Functional dissection of Arabidopsis COP1 reveals specific roles of its three structural modules in light control of seedling development. Embo j 17, 5577–5587 (1998). 10.1093/emboj/17.19.5577 9755158 PMC1170886

[44] Baert J. L. The E3 ubiquitin ligase complex component COP1 regulates PEA3 group member stability and transcriptional activity. Oncogene 29, 1810–1820 (2010). 10.1038/onc.2009.471 20062082

[45] Vitari A. C. COP1 is a tumour suppressor that causes degradation of ETS transcription factors. Nature 474, 403–406 (2011). 10.1038/nature10005 21572435

[46] Xie Y. COP1/DET1/ETS axis regulates ERK transcriptome and sensitivity to MAPK inhibitors. J Clin Invest 128, 1442–1457 (2018). 10.1172/JCI94840 29360641 PMC5873878

[47] Su C.-H. Nuclear export regulation of COP1 by 14-3-3σ in response to DNA damage. Molecular Cancer 9, 243 (2010). 10.1186/1476-4598-9-243 20843328 PMC2949799

[48] Yoon S. H. A parathyroid hormone/salt-inducible kinase signaling axis controls renal vitamin D activation and organismal calcium homeostasis. J Clin Invest (2023). 10.1172/JCI163627 PMC10145948 36862513

[49] Dwivedi P. P., Omdahl J. L., Kola I., Hume D. A. & May B. K. Regulation of rat cytochrome P450C24 (CYP24) gene expression. Evidence for functional cooperation of Ras-activated Ets transcription factors with the vitamin D receptor in 1,25-dihydroxyvitamin D(3)-mediated induction. J Biol Chem 275, 47–55 (2000). 10.1074/jbc.275.1.47 10617584

[50] Li X., Zheng W. & Li Y. C. Altered gene expression profile in the kidney of vitamin D receptor knockout mice. Journal of Cellular Biochemistry 89, 709–719 (2003). 10.1002/jcb.10547 12858337

[51] Lee S. M. Kidney deletions of Cyp27b1 fail to reduce serum 1,25(OH). J Steroid Biochem Mol Biol 250, 106734 (2025). 10.1016/j.jsbmb.2025.106734 40096920 PMC12496027

[52] Meyer M. B., Lee S. M., Cichanski S. R., Cobice D. F. & Pike J. W. Spatial detection and consequences of nonrenal calcitriol production as assessed by targeted mass spectrometry imaging. JCI Insight (2024). 10.1172/jci.insight.181763 PMC11383599 38916957

[53] Pop M. S. A small molecule that binds and inhibits the ETV1 transcription factor oncoprotein. Mol Cancer Ther 13, 1492–1502 (2014). 10.1158/1535-7163.MCT-13-0689 24737027 PMC4154495

[54] Oh S., Shin S., Lightfoot S. A. & Janknecht R. 14-3-3 proteins modulate the ETS transcription factor ETV1 in prostate cancer. Cancer Res 73, 5110–5119 (2013). 10.1158/0008-5472.Can-13-0578 23774214 PMC3745528

[55] Yoon S. H. A parathyroid hormone/salt-inducible kinase signaling axis controls renal vitamin D activation and organismal calcium homeostasis. J Clin Invest 133 (2023). 10.1172/JCI163627 PMC10145948 36862513

[56] Kuro-O M. Mutation of the mouse klotho gene leads to a syndrome resembling ageing. Nature 390, 45–51 (1997). 10.1038/36285 9363890

[57] Shekhar A. Transcription factor ETV1 is essential for rapid conduction in the heart. J Clin Invest 126, 4444–4459 (2016). 10.1172/JCI87968 27775552 PMC5127680

[58] Shekhar A. ETV1 activates a rapid conduction transcriptional program in rodent and human cardiomyocytes. Sci Rep 8, 9944 (2018). 10.1038/s41598-018-28239-7 29967479 PMC6028599

[59] Yamaguchi N. Cardiac Pressure Overload Decreases ETV1 Expression in the Left Atrium, Contributing to Atrial Electrical and Structural Remodeling. Circulation 143, 805–820 (2021). 10.1161/CIRCULATIONAHA.120.048121 33225722 PMC8449308

[60] Janosevic D. The orchestrated cellular and molecular responses of the kidney to endotoxin define a precise sepsis timeline. Elife 10 (2021). 10.7554/eLife.62270 PMC7810465 33448928

[61] Hao Y. Dictionary learning for integrative, multimodal and scalable single-cell analysis. Nat Biotechnol 42, 293–304 (2024). 10.1038/s41587-023-01767-y 37231261 PMC10928517

[62] Hao Y. Integrated analysis of multimodal single-cell data. Cell 184, 3573–3587 e3529 (2021). 10.1016/j.cell.2021.04.048 34062119 PMC8238499

[63] Germain P. L., Lun A., Garcia Meixide C., Macnair W. & Robinson M. D. Doublet identification in single-cell sequencing data using scDblFinder. F1000Res 10, 979 (2021). 10.12688/f1000research.73600.2 35814628 PMC9204188

[64] Zhang Y. Model-based analysis of ChIP-Seq (MACS). Genome Biol 9, R137 (2008). 10.1186/gb-2008-9-9-r137 18798982 PMC2592715

[65] Aran D. Reference-based analysis of lung single-cell sequencing reveals a transitional profibrotic macrophage. Nat Immunol 20, 163–172 (2019). 10.1038/s41590-018-0276-y 30643263 PMC6340744

[66] Miao Z. Single cell regulatory landscape of the mouse kidney highlights cellular differentiation programs and disease targets. Nat Commun 12, 2277 (2021). 10.1038/s41467-021-22266-1 33859189 PMC8050063

[67] DennisG.Jr. DAVID: Database for Annotation, Visualization, and Integrated Discovery. Genome Biol 4, P3 (2003).12734009

[68] Huang da W., Sherman B. T. & Lempicki R. A. Systematic and integrative analysis of large gene lists using DAVID bioinformatics resources. Nat Protoc 4, 44–57 (2009). 10.1038/nprot.2008.211 19131956

[69] Clinkenbeard E. L. Conditional Deletion of Murine Fgf23: Interruption of the Normal Skeletal Responses to Phosphate Challenge and Rescue of Genetic Hypophosphatemia. J Bone Miner Res 31, 1247–1257 (2016). 10.1002/jbmr.2792 26792657 PMC4891276

[70] Askar P. The Etv1/Er81 transcription factor coordinates myelination-related genes to regulate Schwann cell differentiation and myelination. Ann Transl Med 10, 875 (2022). 10.21037/atm-22-3489 36110998 PMC9469140

[71] Meyer M. B., Benkusky N. A. & Pike J. W. The RUNX2 cistrome in osteoblasts: characterization, down-regulation following differentiation, and relationship to gene expression. J Biol Chem 289, 16016–16031 (2014). 10.1074/jbc.M114.552216 24764292 PMC4047377

[72] Meyer M. B. A kidney-specific genetic control module in mice governs endocrine regulation of the cytochrome P450 gene. J Biol Chem 292, 17541–17558 (2017). 10.1074/jbc.M117.806901 28808057 PMC5655528

[73] Heinz S. Simple combinations of lineage-determining transcription factors prime cis-regulatory elements required for macrophage and B cell identities. Mol Cell 38, 576–589 (2010). 10.1016/j.molcel.2010.05.004 20513432 PMC2898526

[74] Zhang Y. Model-based analysis of ChIP-Seq (MACS). Genome Biol 9, R137 (2008). 10.1186/gb-2008-9-9-r137 18798982 PMC2592715

[75] Robinson M. D., McCarthy D. J. & Smyth G. K. edgeR: a Bioconductor package for differential expression analysis of digital gene expression data. Bioinformatics 26, 139–140 (2010). 10.1093/bioinformatics/btp616 19910308 PMC2796818

[76] Anders S. & Huber W. Differential expression analysis for sequence count data. Genome Biol 11, R106 (2010). 10.1186/gb-2010-11-10-r106 20979621 PMC3218662

[77] Ni P. Targeting fibroblast growth factor 23-responsive pathways uncovers controlling genes in kidney mineral metabolism. Kidney Int 99, 598–608 (2021). 10.1016/j.kint.2020.10.024 33159963 PMC7914218

[78] Gibson D. G. Enzymatic assembly of DNA molecules up to several hundred kilobases. Nat Methods 6, 343–345 (2009). 10.1038/nmeth.1318 19363495

