# Supplementary material for: ETV1 Couples Reciprocal FGF23 and PTH Signaling to Renal CYP24A1 Regulation
Source: Res Sq. 2026 Aug 6:rs.3.rs-10474610. Preprint. [Version 1] doi: 10.21203/rs.3.rs-10474610/v1 (PMC13484825; doi:10.21203/rs.3.rs-10474610/v1)
Supplement: Supplementary file 1 [file NIHPPRS10474610V1-supplement-1.pdf]

Supplemental Data Figures

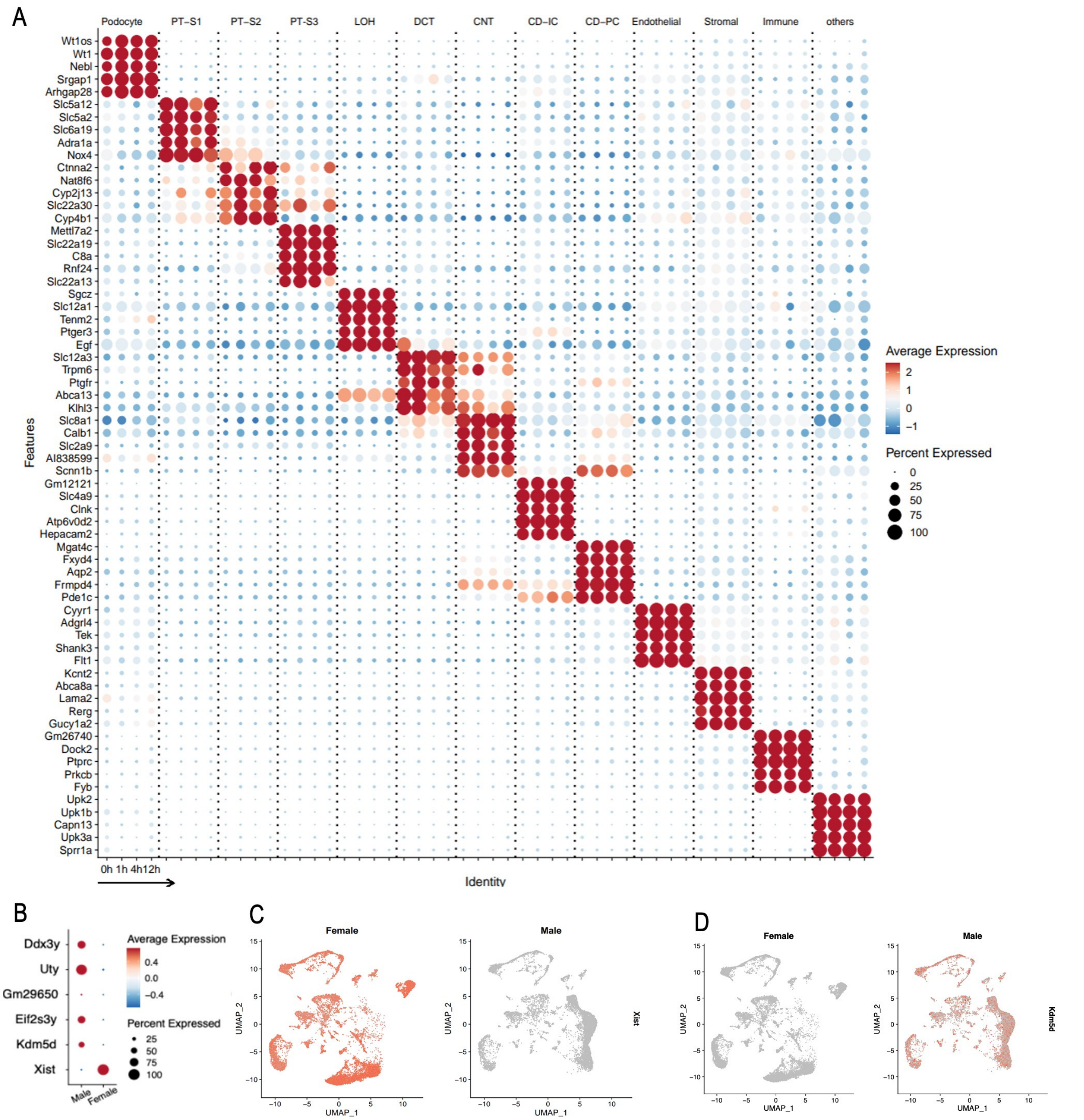

Supplemental Figure 1. Kidney Multiome sex-hashing and hallmark nephron cell markers. A, dot plot of representative nephron-cell specific hallmark gene expression with time (0 h (control, and 1, 4, and 12 h); scale fo6r average expression and percent of cells expressing a specific marker are shown on the far right. **B,** dot plot confirming male- versus female-specific gene expression in the kidney Multiome data set from **Figure 1**; *Xist* was detected only in female mice whereas Y chromosome markers DEAD-box helicase

3 Y-linked (*Ddx3y*), ubiquitously transcribed tetratricopeptide repeat containing, Y-linked (*Uty*), *Gm29650,* eukaryotic translation initiation factor 2, subunit 3, structural gene Y-linked (*Eif2s3y*), lysine-specific demethylase 5D (*Kdm5d*), were detected in males; scale for average expression and percent of cells expressing a specific marker are shown on the far right. **C,** feature plot showing distribution of female-specific *Xist* gene across all cell clusters (left, female; right, male). **D,** feature plot showing male-specific *Kdmd5d* gene expression all cell clusters (left, female; right, male).

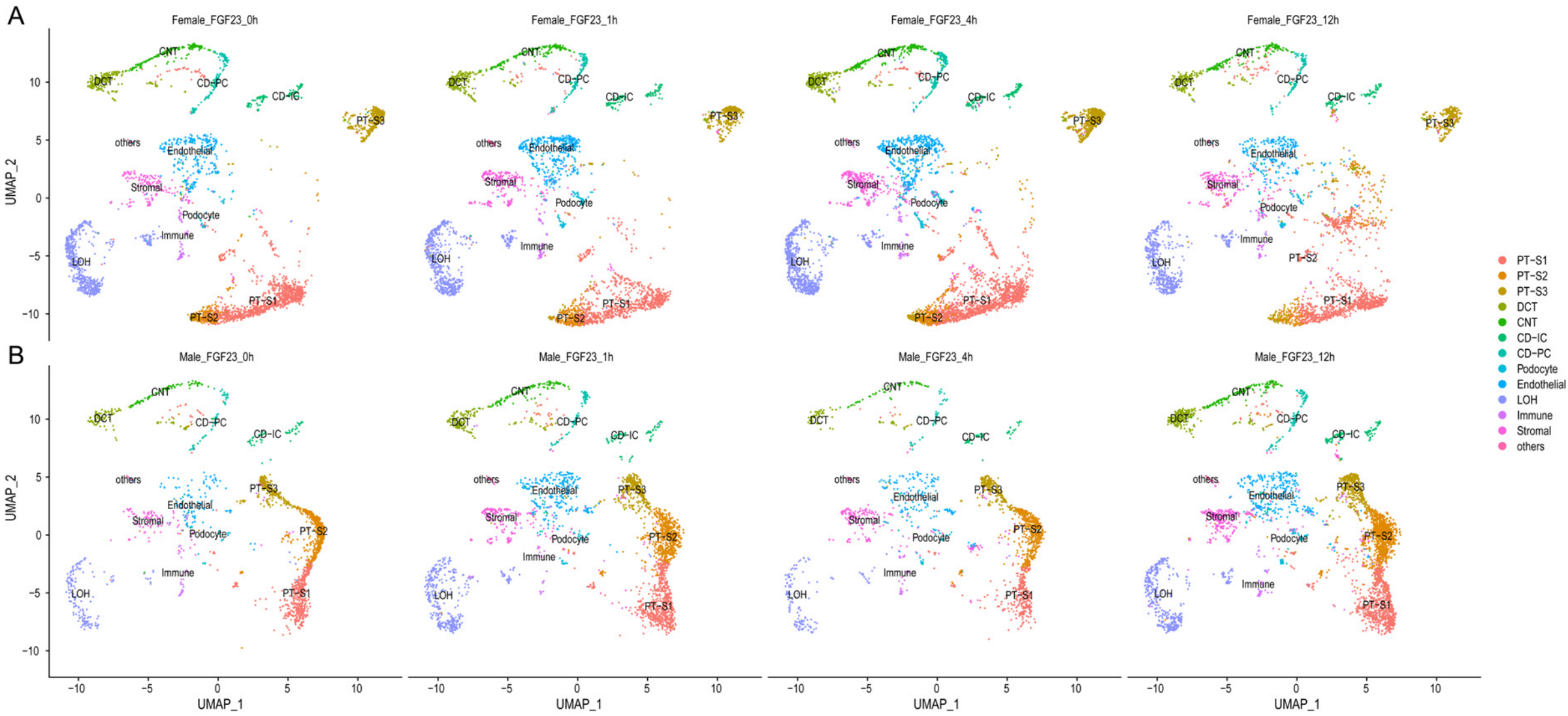

Supplemental Figure 2. Cell distribution by time point. A, female, and **B,** male UMAPs showing cell distributions for the kidney 10X Multiome samples from **Figure 1** at 0, 1, 4, and 12 h. Cell distributions were not affected by FGF23 injections with time.

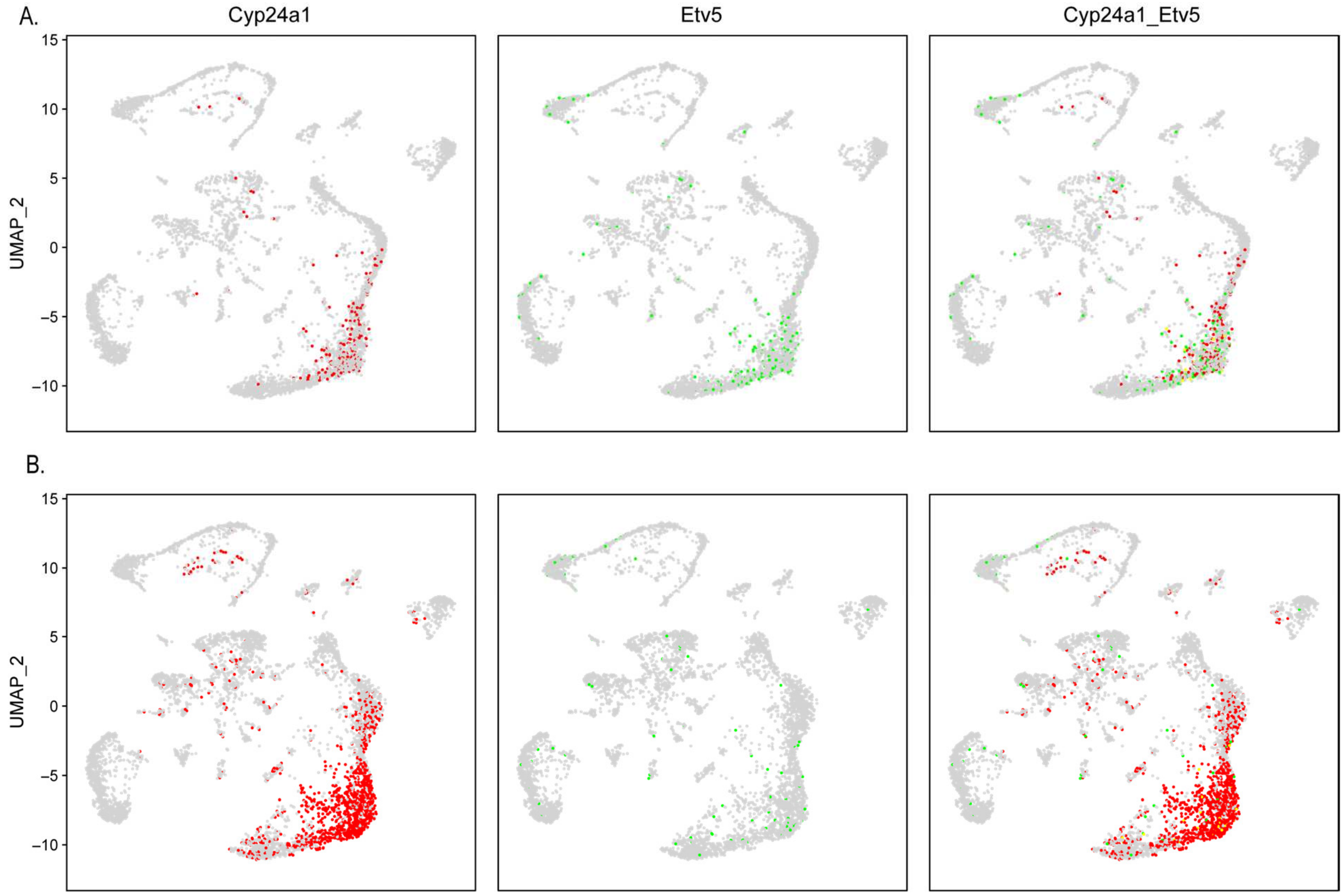

Supplemental Figure 3. *Etv5* co-feature plots from 10X Multiome. Feature plots of *Cyp24a1* (red) and *Etv5* (green) with *co*-expression (yellow) in kidney proximal tubule cells at 0 h (upper row) and 1 h FGF23 injection (lower) from the 10X Multiome dataset (see **Figure 1**).

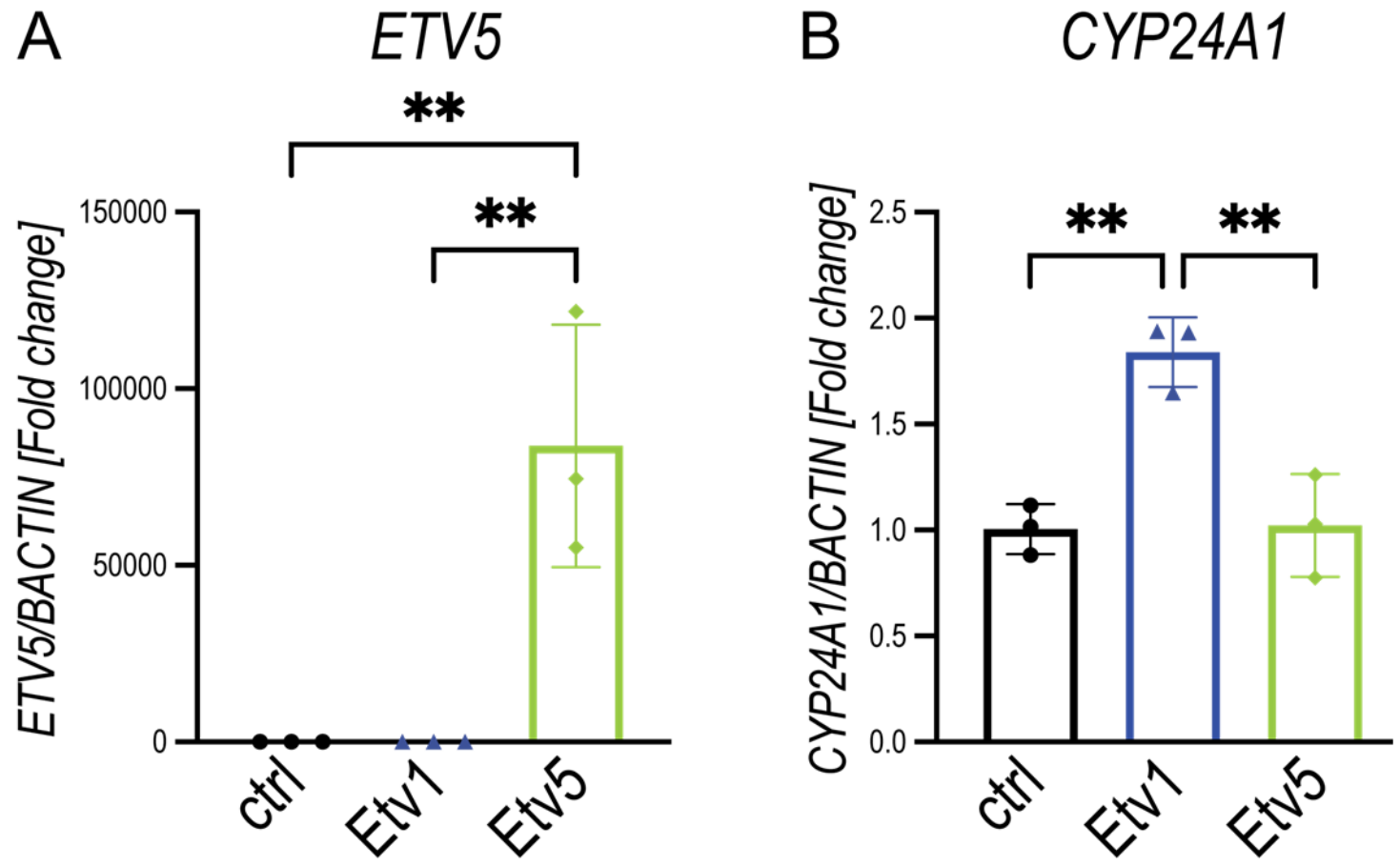

Supplemental Figure 4. ETV5 overexpression was not associated with increased *CYP24A1*. A, versus a GFP control (ctrl) cDNA plasmid, ETV5-FLAG cDNA transfected HEK-mKL cells showed marked induction of ETV5 but not ETV1 mRNA as determined by qPCR; **B,** *CYP24A1* mRNA expression after transfection of HEK-mKL cells with the control GFP, ETV1-FLAG, or ETV5-FLAG cDNAs (24 h) as determined by qPCR; ETV1-FLAG increased *CYP24A1* mRNA (**p<0.01; ****p<0.0001).

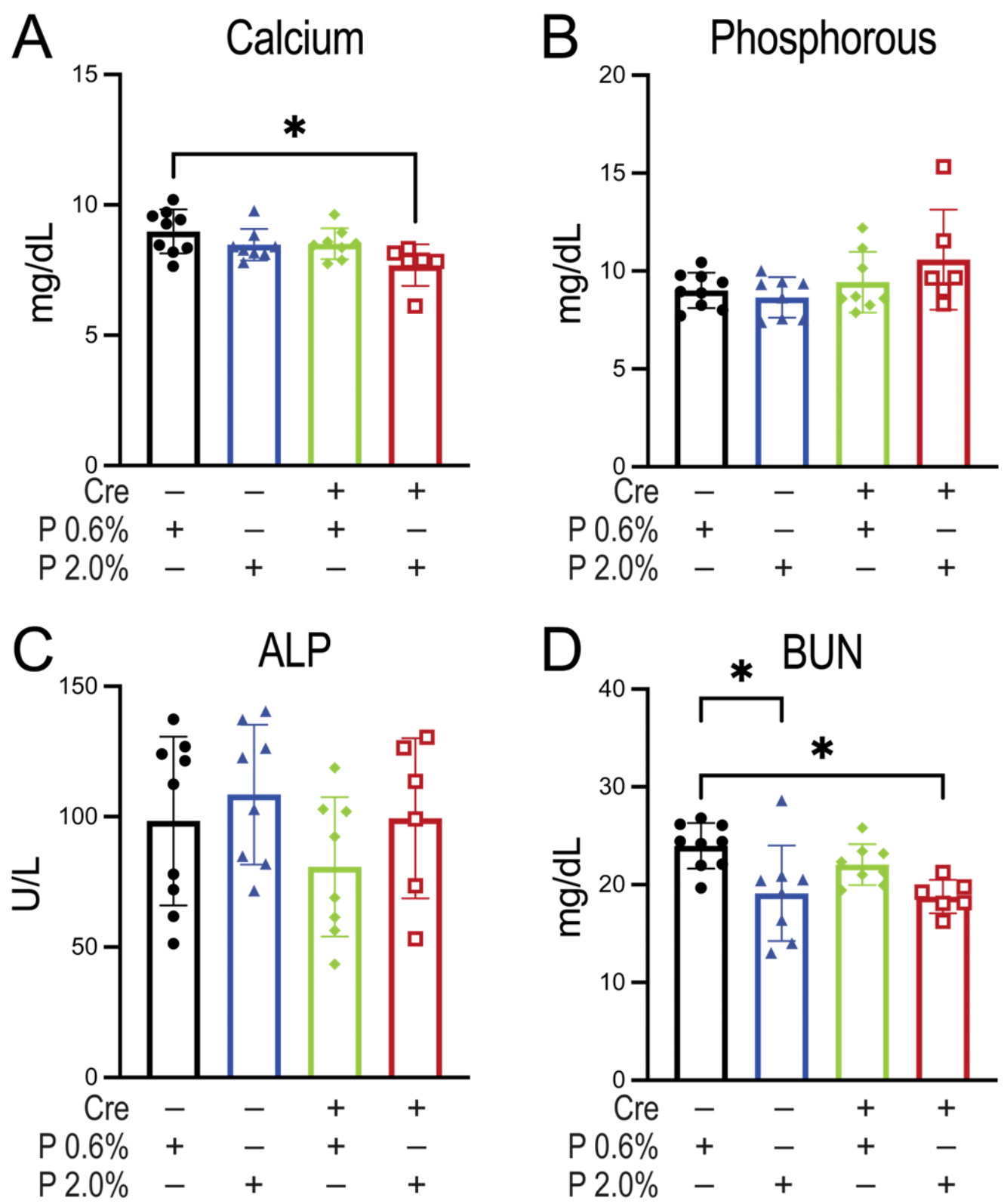

Supplemental Figure 5. Serum biochemistries of mice during high phosphate diet challenge. Serum biochemical analysis from the Etv1$^{fl/fl}$/Pax8-Cre$^{+/-}$ receiving normal P (0.6%; 'NP') or high P (2.0%; 'HP') diet: **A**, calcium, **B,** phosphorus, **C,** alkaline phosphatase (ALP), **D,** blood urea nitrogen (BUN), (n=6/8 group). Data were compared using one-way ANOVA with Tukey post-tests, which showed that Cre^{+} HP diet treated mice had decreased calcium vs. Cre^{-} NP treated mice (*p > 0.05). Cre^{+} HP and Cre^{-} HP treated mice had decreased BUN vs Cre^{-} NP diet treated mice (*p<0.05).

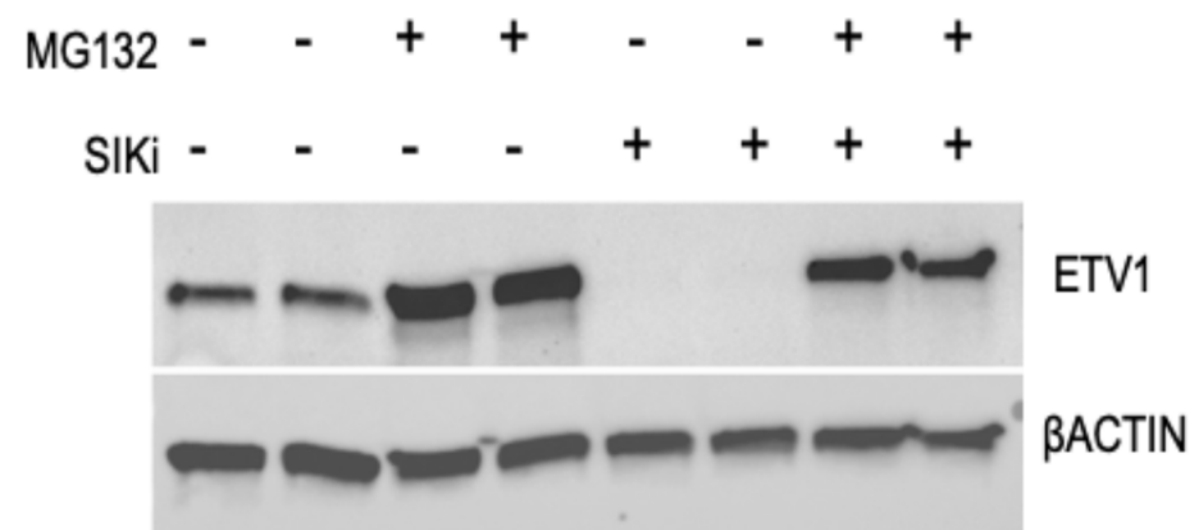

Supplemental Figure 6. Effects of proteosome inhibitor on ETV1 in RPTEC cells. Immunoblot for ETV1 compared to β-ACTIN in whole-cell lysates from human renal proximal tubule epithelial cell (RPTEC) line cells treated with MG132 (5 μM) for 2 h, with and without SIKi (10 μM) for an additional 4 h. Samples were analyzed in duplicate: vehicle (lanes 1-2), MG132 (lanes 3-4); SIKi (5-6), MG132 + SIKi cotreatment (lanes 7-8).

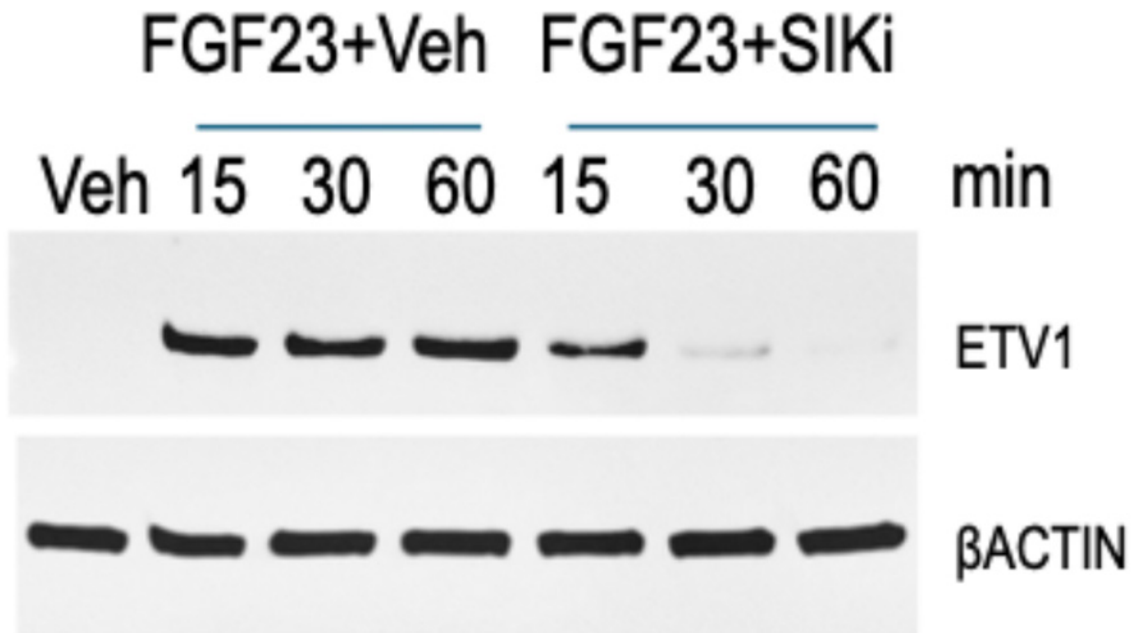

Supplemental Figure 7. Rapid SIKi inhibition of ETV1. Immunoblot of ETV1 protein expression in HEK-mKL cells treated with 50 ng/mL of FGF23 for 8 hours and then SIKi for 15, 30, 60 min; β-ACTIN was probed as a loading control.

Supplemental Table 1: ETV1 occupancy change statistics. Peak regions were evaluated for their normalized, replicate tag density by DESeq2 as indicated in Methods. The log2 fold change (log2FC), P value, and false discovery rate (FDR) are indicated for each genomic region near *Cyp24a1*. *Cyp24a1* exon regions (ex2 and ex5) are included as intergenic controls and are not statistically changed.

Supplemental Table 2: ETV1 peak locations from ChIP-seq data. Chromosome, start coordinate, stop coordinate, peak ID, peak stat, and strand (BED file format) are indicated for ETV1 Veh peaks, ETV1 FGF23 peaks, ETV1 FGF23+PTH peaks, and ETV1 PTH peaks on each tab.

Supplemental Table 3: List of the commercially available human and mouse qPCR primer/probe sets used in the mRNA quantification analyses.

Supplementary Files

This is a list of supplementary files associated with this preprint. Click to download.

- SupplementalTable1.xlsx
- SupplementalTable2.xlsx
- SupplementalTable3.xlsx
